# Stabilization of recurrent neural networks through divisive normalization

**DOI:** 10.1101/2025.05.16.654567

**Published:** 2025-05-21

**Authors:** Flaviano Morone, Shivang Rawat, David J. Heeger, Stefano Martiniani

**Affiliations:** Center for Neural Science, NYU and Center for Soft Matter Research, Department of Physics, NYU; Center for Soft Matter Research, Department of Physics, NYU and Courant Institute of Mathematical Sciences, NYU; Department of Psychology and Center for Neural Science, NYU; Center for Neural Science, NYU Center for Soft Matter Research, Department of Physics, NYU; Courant Institute of Mathematical Sciences, NYU and Simons Center for Computational Physical Chemistry, Department of Chemistry, NYU

## Abstract

Stability is a fundamental requirement for both biological and engineered neural circuits, yet it is surprisingly difficult to guarantee in the presence of recurrent interactions. Standard linear dynamical models of recurrent networks are unreasonably sensitive to the precise values of the synaptic weights, since stability requires all eigenvalues of the recurrent matrix to lie within the unit circle. Here we demonstrate, both theoretically and numerically, that an arbitrary recurrent neural network can remain stable even when its spectral radius exceeds 1, provided it incorporates divisive normalization, a dynamical neural operation that suppresses the responses of individual neurons. Sufficiently strong recurrent weights lead to instability, but the approach to the unstable phase is preceded by a regime of critical slowing down, a well-known early warning signal for loss of stability. Remarkably, the onset of critical slowing down coincides with the breakdown of normalization, which we predict analytically as a function of the synaptic strength and the magnitude of the external input. Our findings suggest that the widespread implementation of normalization across neural systems may derive not only from its computational role, but also to enhance dynamical stability.

## I. INTRODUCTION

**FIG. 1.**
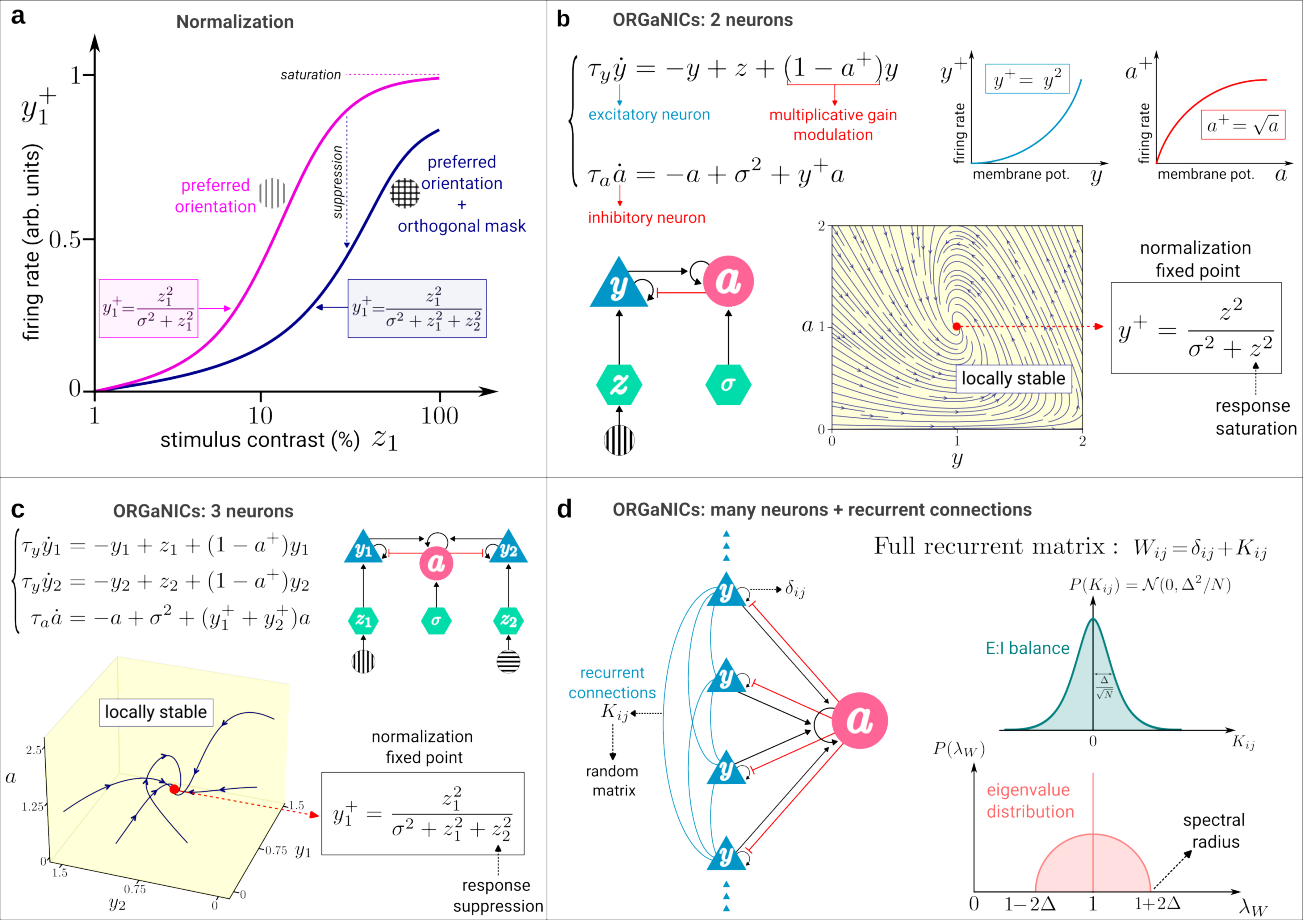
Normalization via ORGaNICs. **a,** An orientation selective principal neuron $y_{1}$ in primary visual cortex (V1) fires when the stimulus orientation matches its preferred orientation (pink curve). The larger the stimulus contrast $z_{1}$, the greater the strength of the response. The firing rate $y_{1}^{+}$ can be modeled by a nonlinear response function that saturates at high contrast. When a second grating stimulus $z_{2}$ with orientation perpendicular to the preferred one (*viz*. an orthogonal mask) is presented simultaneously with stimulus $z_{1}$, there is a rightward shift of the response function $y_{1}^{+}$ (blue curve). The suppressive effect of the orthogonal mask can be modeled by an extra term $z_{2}^{2}$ in the denominator of the response function. **b,** The saturation of the firing rate $y^{+}$ at high contrast z can be obtained as the fixed point of the 2-neuron circuit in Eq. (2) involving the principal neuron y and a secondary inhibitory neuron a that acts on y as a multiplicative gain modulator. This fixed point is locally stable for any value of the time constants $\tau_{y},\;\tau_{a}$ and the semisaturation constant σ. **c,** The suppressive effect of the orthogonal mask can be modeled by the fixed point of the 3-neuron circuit, where $y_{1}$ and $y_{2}$ respond selectively to the vertical and horizontal orientations, respectively, and a performs the multiplicative gain modulation on both $y_{1}$ and $y_{2}$. This fixed point is locally stable for any value of the parameters. Notice that neurons $y_{1}$ and $y_{2}$ do not interact directly, but only through neuron a, i.e., there are no recurrent connections between the principal neurons. **d,** Recurrent connections are included via the weight matrix W composed of the identity I plus a random matrix K modeling lateral synaptic connections between the principal neurons. The weights $K_{ij}$ are E:I balanced (i.e., mean 0) and sampled from a symmetric Gaussian distribution such that the spectral radius of W is equal to 1+2Δ in the limit where the number of neurons N goes to infinity.

Divisive normalization is a form of multiplicative neuronal modulation occurring in the brain whereby the response of an individual neuron is divided by the summed activity of other similarly tuned neurons. Introduced in the 1990s to explain nonlinearities in the responses of neurons in the primary visual cortex [1, 2], it was later invoked to interpret a larger body of physiological data in the olfactory [3] and auditory [4] cortical areas, as well as in cognitive processes such as attention, working memory and value-based decision making [5–8]. Simply put, normalization explains how the response of a given neuron, which is selective for a specific stimulus, is suppressed by different stimuli that would elicit a weaker or no response if they were presented alone, as illustrated in Fig. 1a. Notwithstanding the general character of this neural computation, different biophysical mechanisms may perform normalization in different neural systems, including intracortical shunting inhibition [2, 9], thalamocortical synaptic depression [10], pre-synaptic inhibition [3], recurrent amplification (i.e., amplifying weak inputs more than strong inputs) [11–15], to name the most prominent ones. In our computational model, we implement divisive normalization via a multiplicative interaction between the principal neurons and a population of (secondary) inhibitory neurons, as illustrated in Fig. 1b,c. This model, which goes by the name of ORGaNICs [16], is much like the linear recurrent circuits introduced in the 1980s [17], but with a multiplicative gain on the recurrent term that implements normalization (see Fig. 1b,c).

ORGaNICs have been proved to be unconditionally stable when the recurrent weight matrix is the identity [18]. Here we elaborate on the relationship between normalization and stability in the case of a generic recurrent weight matrix, such as a large random matrix drawn from the Gaussian Orthogonal Ensemble (GOE) [19]. We find that ORGaNICs models with recurrent weights drawn from the GOE ensemble are stable even when the spectral radius of the recurrent matrix is larger than 1, thanks to the normalization mechanism. Quantitatively, ORGaNICs push the stability limit of linear models by more than 100% (see phase diagram of stability in Fig. 5). Perhaps more importantly, we find that the transition to an unstable fixed point is preceded by critical slowing down [20, 21] in the neural dynamics (where the circuit is slow to reach the fixed point or to recover from small perturbations), the onset of which co-occurs with the breakdown of normalization in the neural responses. Remarkably, this result implies that the breakdown of normalization is an early warning signal for the loss of stability of the neural network, a signal we can predict analytically in terms of the recurrent interaction strength (i.e., the variance of the recurrent weights) and the magnitude of the external input.

## II. ORGaNICs MODEL OF NORMALIZATION

In its simplest form, normalization works by dividing a neuron’s total input by the sum of all inputs to N neurons in the normalization pool [1], expressed mathematically by the formula

(1) $$y_{i}^{+}=\frac{z_{i}^{2}}{\sigma^{2}\;+\;\sum_{j=1}^{N}z_{j}^{2}},$$

where $y_{i}^{+}$ is the firing rate of neuron $i;z_{i}\in [0,\;1]$ is its input drive, defined as a weighted sum of the responses of a population of presynaptic neurons; and σ is the semisaturation constant, whose experimental value in primary visual cortex (V1) is σ∼0.1 [22]. The purpose of the normalization mechanism is to normalize the output responses $y_{i}^{+}$ via the ratio between the input drive of an individual neuron and the input drives summed across all of the neurons [5, 16, 23–29]. Two important predictions of the normalization equation (1) as applied to visual cortex are illustrated in Fig. 1a, namely response saturation and cross-orientation suppression.

Since Eq. (1) describes a neural process that is static, it is natural to ask how the output responses $y_{i}^{+}(t)$ evolve in time towards the normalized state given by Eq. (1). That is: how does a neural circuit accomplish normalization? A mathematical way to achieve normalization dynamically is to couple the output responses of the principal neurons, $y_{i}^{+}(t)$, to a secondary neuronal population, represented by a single variable a(t), that acts as a multiplicative inhibitory modulator. The class of dynamical systems implementing divisive normalization in this way is known as ORGaNICs [16, 18]. The simplest ORGaNICs involve only two neurons and is described by the following dynamical equations

(2) $$\left\{\begin{array}{l} \tau_{y}\dot{y}=-y+z+\left(1-a^{+}\right)y\\ \tau_{a}\dot{a}=-a+\sigma^{2}+y^{+}a\;, \end{array}\right.$$

where y(t) and a(t) represent the membrane potentials (relative to an arbitrary threshold potential that we take to be 0) of the excitatory (E) and inhibitory (I) neurons, respectively, and $y^{+}$ and $a^{+}$ are the corresponding firing rates. The 2-neuron circuit described by Eq. (2) is depicted in Fig. 1b. The firing rate of the E neuron $y^{+}$ is related to the membrane potential by squaring, $y^{+}=ky^{2}$ [1, 30–33] (henceforth we set the dimensional proportionality factor k=1), while the firing rate of the I neuron is given by $a^{+}=\sqrt{\left\lfloor a\right\rfloor }$, where ⌊x⌋=max(0,x) (see Supplementary Section V E and Fig. S10 for alternative activation functions); and $\tau_{y}>0,\tau_{a}>0$ are the neurons’ intrinsic time constants. The circuit in Eq. (2) models, for example, the response of a neuron in the primary visual cortex with z proportional to stimulus contrast, as seen in Fig. 1b. At the fixed point, the principal neuron y follows the normalization equation (1), i.e. $y^{+}=\frac{z^{2}}{\sigma^{2}+z^{2}}$, which explains the *saturation* of the firing rate at large contrast z. Moreover, this normalization fixed point is always locally stable [18] (see Fig. 1b, c).

Although it has been proved that a two-neuron ORGaNICs is unconditionally stable for any strength of the recurrent drive [18], the stability of a high-dimensional circuit with arbitrary recurrent connections has not been studied. Thus we ask: what happens when arbitrary recurrent connections (i.e. interactions) are included in the circuit? Do ORGaNICs still accomplish normalization? Is stability preserved?

To answer these questions we include recurrent connections between the principal neurons as described by the following set of differential equations

(3) $$\left\{\begin{array}{l} \tau_{y}\dot{y_{i}}=-y_{i}+z_{i}+\left(1-a^{+}\right)\sum_{j=1}^{N}W_{ij}y_{i}\\ \tau_{a}\dot{a}=-a+\sigma^{2}+\left(\sum_{i=1}^{N}y_{i}^{+}\right)\;a, \end{array}\right.$$

where the recurrent weight matrix W captures lateral connections between the principal neurons, as shown in Fig. 1d. Our goal is twofold: first, we determine the conditions on W and z such that normalization still approximately holds for the circuit in Eq. (3); second, we investigate the consequences of the breakdown of normalization, due to strong recurrent interactions, for the stability of the whole neural network.

## III. LOSS OF NORMALIZATION AS AN EARLY WARNING SIGNAL OF NEURODYNAMICAL INSTABILITY

### A. Numerical solution of the fixed point

We start with a numerical study of the stability of the fixed-point of Eq. (3) and then we derive our analytical solution perturbatively, supported by the exact numerical result. We express the recurrent matrix as the sum of the identity plus a perturbation as

(4) W=I+K,

where K is a symmetric GOE random matrix [19] whose entries $K_{ij}$ are independent and identically distributed Gaussian random variables with zero mean and variance $\Delta^{2}/N$ if i=j or $\Delta^{2}/2N$ if i≠j, corresponding to balanced excitation and inhibition. The scaling of the variance with 1/N ensures that the spectral radius ρ(K) does not grow with the number of neurons N, but is controlled only by Δ (specifically, ρ(K)=2Δ, hence ρ(W)=1+2Δ, see Fig. 1d). The choice of a random matrix to study the stability of large systems of differential equations can be traced back to the seminal work of May on the stability of complex ecosystems [34], that initiated a new field in theoretical ecology [35, 36] as well as the famous diversity-stability debate [37–39]. Methods based on random matrix theory are also well suited to model very large neural circuits whose experimental parametrization would be otherwise unfeasible [40]. Here, we follow a similar approach with the goal of deriving a condition on the recurrent interaction strength Δ such that the output responses $y_{i}$ still approximately satisfy the normalization equation (1), and then study the consequences of the breakdown of normalization on the system’s stability. We note, *en passant*, that a linear model (i.e. a model where a≡0) would become unstable as soon as the spectral radius of W gets larger than 1, i.e. a soon as Δ>0 (see Fig. 5). In contrast, the nonlinear model described by Eq. (3) can be stable even when W has spectral radius larger than 1, as we show next.

**FIG. 2.**
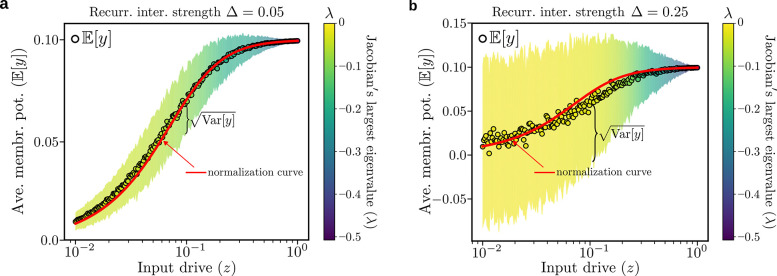
Numerical solution to the ORGaNICs’ fixed point equations. **a,** Fixed-point average membrane potential E[y] (open circles) as a function of the input drive z (here we use N=100 neurons and $z_{i}=z/\sqrt{N}$, called *delocalized* input drive) for an E:I balanced recurrent network K with zero mean and std. dev. Δ=0.05, obtained by solving numerically Eq. (3) using the explicit Euler method with time step $dt=0.05\times \tau_{y}$. The semisaturation constant is σ=0.1 and the neurons’ time constants $\tau_{y}$ and $\tau_{a}$ are equal. Each point is an average over 1000 realizations of the synaptic weight matrix K. The neural response still follows, on average, the normalization Eq. (1) (solid red curve), but picks up a variance across different random samples of recurrent synaptic weights, represented by the shaded area around the data points. The color code of the shaded area represents the real part of the largest eigenvalue of the Jacobian at the fixed point averaged over samples, whose value is well below 0 for all z. **b,** For Δ=0.25 the average response is still normalized, but the variance (across random samples of the recurrent weights) is bigger than in (**a**) (see Fig. S1 for more Δ values). For sufficiently small input drives, the largest eigenvalue of the Jacobian at the fixed point becomes very small (λ∼0). As a consequence, convergence to the fixed point occurs on time scales much longer than the time constant $\tau_{y}$ of individual neurons, a phenomenon known as critical slowing down (see also Fig. 3c).

In Figure 2a,b we show the numerical solution of the fixed point of Eq. (3) (see Supplementary Section V A for details on the numerical methods). We plot the mean and variance of the fixed point membrane potentials, $y_{i}$, over the ensemble of random recurrent matrices K, as a function of the input drive z for Δ=0.05 and Δ=0.25. We find that the output responses, on average, still follow the normalization curve, i.e. $E\left[y_{i}\right]∼z_{i}/\sqrt{\sigma^{2}+{\left\|z\right\|}^{2}}$ (noting that membrane potential in this model is the square root of firing rate), but pick up a variance that increases with increasing Δ. For sufficiently large Δ we observe that the circuit’s convergence to its fixed point, as measured by the real part of the largest eigenvalue λ of the Jacobian evaluated at the fixed point [41], becomes very slow (i.e. λ∼0 corresponding to a convergence time $t_{\mathrm{conv}}=\frac{1}{\left|\lambda \right|}\gg 1$), as seen in Fig. 2b. This phenomenon, called **critical slowing down**, is widely considered to be an important early warning signal that anticipates the system’s tipping point [20, 21, 42].

**FIG. 3.**
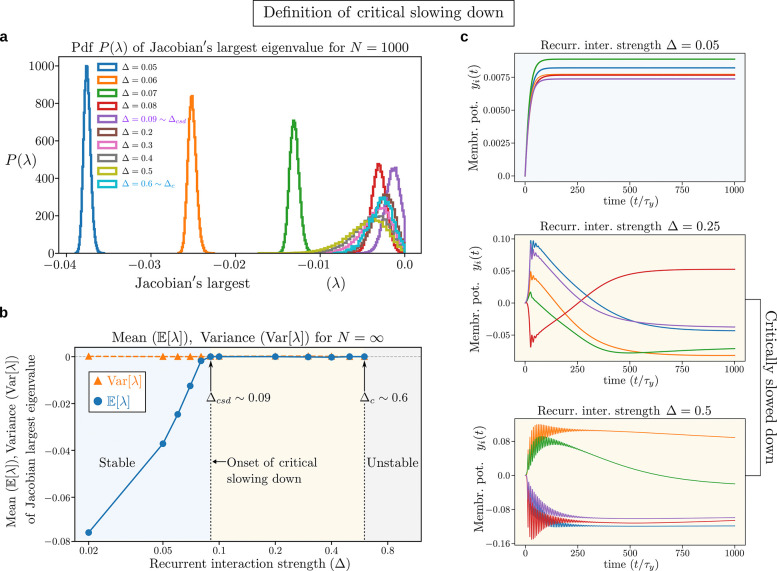
Definition of critical slowing down. **a,** Probability distribution of the largest eigenvalue of the Jacobian at the fixed point for a network with N=1000 neurons (see Fig. S2 for different sizes); input drive $z_{i}=0.01/\sqrt{N}$ (very weak input drive); semisaturation constant σ=0.1; and several values of the recurrent interaction strength Δ. For small Δ the distribution P(λ) is gapped from 0 and, as a consequence, the neural responses converge quickly to their fixed points. When Δ increases, the gap shrinks and then closes at $\Delta =\Delta_{\text{csd}}$, signaling the onset of critical slowing down. For $\Delta_{csd}\leq \Delta \leq \Delta_{c}$ the neural responses converge slowly to their fixed points. For $\Delta \geq \Delta_{c}$ the neural circuits do not have stable fixed points, but exhibit limit cycles and, for even larger Δ, they eventually become unstable (see Fig. 5). **b,** Mean, E[λ], and variance, Var(λ), of the largest eigenvalue of the Jacobian extrapolated to N→∞ as a function of Δ (see Fig. S3 for details on the extrapolation). The model parameters’ values are as in (**a**). Since the variance is zero in the N→∞ limit, P(λ) tends to a delta function δ(λ-E[λ]), thus making the determination of $\Delta_{csd}$ well defined as the value at which the mean E[λ] goes to zero. Slowing down persists up to the critical value $\Delta_{c}$, beyond which there are no stable fixed points (see Fig. 5). **c,** Representative trajectories of the neural responses $y_{i}(t)$ in the stable phase (Δ=0.05) and in the critically slowed down phase (Δ=0.25,0.5), showing the slowness of the dynamics in reaching the fixed point. (see Fig. S4 for trajectories of all the neurons and Figs. S9, S12 for the analysis of the frequency of oscillations).

To identify the onset of critical slowing down we plot in Fig. 3a the probability distribution P(λ) of the real part of the largest eigenvalue of the Jacobian at the fixed point for circuits with N=1000 neurons, weak input drive z=0.01, and different values of the recurrent interaction strength Δ. At small Δ,P(λ) has a gap from 0, which closes when Δ approaches the critical value $\Delta =\Delta_{csd}$, signaling the onset of critical slowing down. To get a more precise estimate of $\Delta_{csd}$, we extrapolate the mean and variance of P(λ) in the limit N→∞ via finite size analysis, yielding the asymptotic mean and variance shown in Fig. 3b (see Supplemenatry Section V B and Figs. S2, S3 for details on the extrapolation to N→∞). The variance goes to zero in the large N limit, meaning that P(λ) becomes a δ-function sharply peaked around its mean. The mean vanishes at $\Delta =\Delta_{csd}$ and remains zero in the whole interval $\Delta_{csd}\leq \Delta \leq \Delta_{c}$ (Fig. 3b). In this interval the neural dynamics are very slow (compared to the neuron’s intrinsic time scale $\tau_{y}$) to reach the fixed point, as illustrated by some representative trajectories shown in Fig. 3c. Eventually, for $\Delta \geq \Delta_{c}$, the circuits enter first into limit cycles and then become unstable (see Fig. 5).

Next we demonstrate that the onset of critical slowing down occurs precisely when normalization of the neural responses breaks down.

### B. Loss of normalization predicts critical slowing down

**FIG. 4.**
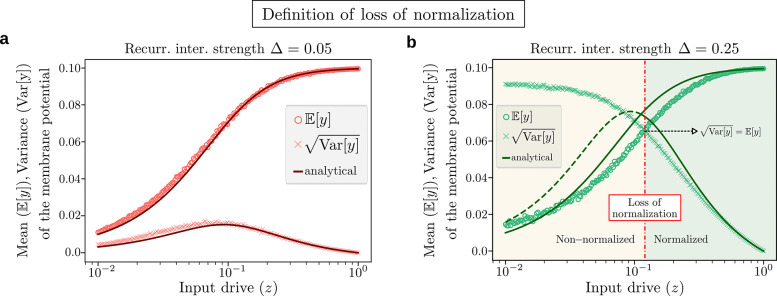
Definition of loss of normalization. **a,** Mean, E[y], (circles) and standard deviation, $\sqrt{Var[y]}$, (crosses) of the fixed point membrane potential as a function of the norm z of the input drive $z_{i}=z/\sqrt{N}$ for an E:I balanced recurrent network K with zero mean and std. dev. Δ=0.05. We used N=100 neurons and averaged over 10^4^ realizations of K. The standard deviation is smaller than the mean for all values of z, so the neural responses are always normalized. The analytical approximations (solid curves) for the mean and standard deviation Eq. (7) of the response, computed with perturbation theory, show a good agreement with the exact numerical solutions. **b,** Same as in **a**, but using Δ=0.25. The standard deviation is smaller than the mean $\left(\sqrt{Var[y]}<E[y]\right)$ at large z, but it is larger than the mean $\left(\sqrt{Var[y]}>E[y]\right)$ at small z. The value of z where the two curves cross each other, given by Eq. (5), defines the threshold at which the neural responses lose normalization (dashed red line). The analytical approximations are in good agreement with numerical simulations for almost all values of the input drive (notice the log scale on the abscissa), but become less accurate at small z where the responses are non-normalized and perturbation theory breaks down, another indication of a major shift in the circuit’s behavior.

**FIG. 5.**
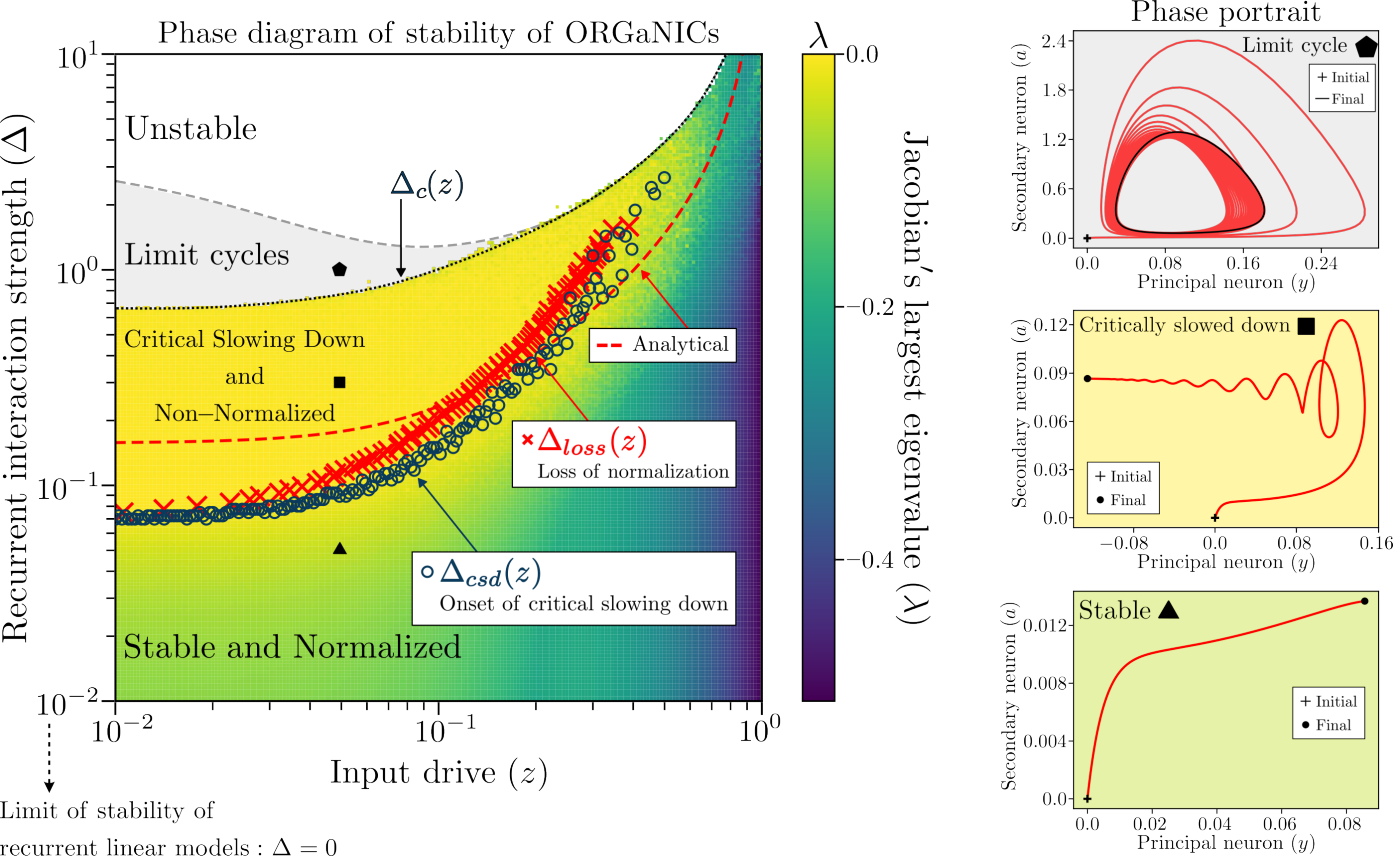
Loss of normalization predicts the onset of critical slowing down. Real part of the largest eigenvalue λ of the Jacobian at the fixed point in the (z,Δ) plane obtained by solving numerically Eq. (3) for E-I balanced networks with N=100 neurons (see Supplementary Section V F and Fig. S11 for the case of E-I imbalanced networks); delocalized input drive $z_{i}=z/\sqrt{N}$; semisaturation constant σ=0.1; neurons’ time constants $\tau_{y}=\tau_{a}$; using a mesh of 200 × 200 values of z and Δ. Color represents the maximum value of λ across 100 random samples of the recurrent synaptic weights. Circuits with small Δ are stable at any value of the input drive z and converge quickly to their fixed point, as indicated by a strictly negative eigenvalue λ<0 and by the *Stable* phase portrait in the (y,a) plane (where a is the inhibitory neuron). Conversely, for $\Delta_{csd}\leq \Delta <\Delta_{c}$, the circuits exhibit critical slowing down, in that they approach the fixed point very slowly, as indicated by λ∼0 and by the spiral attractor in the *Critically slowed down* phase portrait. The points marking the onset of critical slowing down (open blue circles) are determined by the closing of the gap in the distribution P(λ) (see Fig. 3), which define the curve $\Delta_{csd}(z)$. The points defining $\Delta_{\mathrm{loss}}(z)$ (red crosses) represent the boundary between the normalized and non-normalized phases and are determined via Eq. (5) (see Fig. 4). Loss of normalization predicts well the onset of critical slowing down, i.e. $\Delta_{\mathrm{loss}}(z)\approx \Delta_{\mathrm{csd}}(z)$, thus providing a good early warning indicator of neurodynamical tipping points. For sufficiently large Δ the neural circuits exhibit limit cycles, as shown in the *Limit cycle* phase portrait, and for even larger Δ they become unstable. Notice, however, that simple linear recurrent models would become unstable as soon as Δ>0, while adding normalization pushes the stability limit much further.

To quantify the loss of normalization, we look at the mean and variance (across many instances of the recurrent matrix K) of the neural responses. As seen in Figure 2, the neural response $y_{i}$ can be described by its expected value plus the standard deviation: the expected value follows the normalization equation and the standard deviation quantifies the departure from normalization. Therefore, neuron i loses normalization as soon as the standard deviation of its response is equal to its mean value, as given by the formula

(5) $$\sqrt{Var\left[y_{i}\right]}=E\left[y_{i}\right]\;(\text{loss}\;\text{of}\;\text{normalization})\;,$$

and illustrated in Fig. 4a,b. Equation (5) defines implicitly a threshold $\Delta_{\mathrm{loss}}(z)$ marking the boundary between the phase in which responses are normalized and the phase where they are not. We compare the loss of normalization threshold $\Delta_{\mathrm{loss}}(z)$ with the critical slowing down threshold $\Delta_{csd}(z)$ in Fig. 5, showing excellent agreement between the two at all values of the input drive z, thus demonstrating that the onset of critical slowing down co-occurs with the loss of normalization of the neural responses, which represents our most important result.

An immediate consequence of this correspondence is that we can predict theoretically the onset of critical slowing down by calculating $\Delta_{\text{loss}}(z)$, which is a simpler quantity to estimate analytically, as explained next. To compute the mean and variance entering in equation (5), we must first find the fixed point of the dynamical system in Eq. (3), which, unfortunately, cannot be expressed in closed form. To overcome this obstacle, we use perturbation theory to approximate the exact solution. We look for a solution to the fixed point equations in the form of a series $y_{i}=y_{i}^{\left(0\right)}+y_{i}^{\left(1\right)}+\ldots$, where $y_{i}^{\left(0\right)}$ is given by the normalization equation (1), and $y_{i}^{\left(1\right)}$ is of the same order of magnitude as the perturbation K. Inserting this expansion in Eq. (3) we find the approximate fixed point solution (see details in Supplementary Section V C):

(6) $$y_{i}\approx \frac{z_{i}}{\sqrt{\sigma^{2}\;+\;{\left\|z\right\|}^{2}}}+\left(\sum_{j}K_{ij}z_{j}-z_{i}\frac{z^{T}Kz}{\sigma^{2}+{\left\|z\right\|}^{2}}\right)G\left(\left\|z\right\|\right),$$

where $G(z)=\frac{1-\sqrt{\sigma^{2}+z^{2}}}{\sigma^{2}+z^{2}}$ and ${\left\|z\right\|}^{2}=z^{T}z$. The last term on the right hand side of Eq. (6) quantifies the impact of the recurrent interactions on the normalization fixed point. Taking the expectation on both sides, and using the fact that the $K_{ij}$’s have zero mean, we find $E\left[y_{i}\right]\approx \frac{z_{i}}{\sqrt{\sigma^{2}+{\left\|z\right\|}^{2}}}$, meaning that the neural responses still follow, on average, the normalization equation, as seen in Figure 4a,b. Departure from normalization is quantified by the variance of $y_{i}$. The calculation of $Var\left[y_{i}\right]$ yields the following general expression (see Supplementary Section V C for details)

(7) $$Var\left[y_{i}\right]\approx \frac{\Delta^{2}}{2N}\left[{\left\|z\right\|}^{2}-z_{i}^{2}+\frac{2z_{i}^{2}\sigma^{4}}{{\left(\sigma^{2}\;+\;{\left\|z\right\|}^{2}\right)}^{2}}\right]G^{2}\left(\left\|z\right\|\right)\;,$$

which depends on the magnitude ‖z‖ and shape $z_{i}$ of the input drive. For example, we consider a *delocalized* input drive, i.e. $z_{i}=\frac{z}{\sqrt{N}}$, (the opposite case of a *localized* input drive is discussed in Supplementary Section VC2 and Figs S6, S7, S8, leading to qualitatively similar results) and find that $Var\left[y_{i}\right]\approx \frac{\Delta^{2}z^{2}}{2N}G^{2}(z)$, independent of i. In Figure 4a,b we plot the mean and variance of the neural response for two values of Δ, showing that the analytical approximations agree well with the exact numerical solution (see Fig. S5 for more values of Δ). Finally, by equating the mean and the standard deviation of the response, we find the threshold $\Delta_{\mathrm{loss}}(z)$ marking the boundary between the normalized and non-normalized phases as

(8) $$\Delta_{loss}\left(z\right)=\frac{\sqrt{2\left(\sigma^{2}\;+\;z^{2}\right)}}{1\;-\;\sqrt{\sigma^{2}\;+\;z^{2}}}\;,$$

The analytical approximation given by Eq. (8) for the function $\Delta_{\mathrm{loss}}(z)$, shown in Fig. 5, is in good agreement with the exact numerical estimate. Since $\Delta_{\mathrm{loss}}(z)\approx \Delta_{csd}(z)$, Eq. (8) can be used to predict the onset of critical slowing down in the neural dynamics from the magnitude of the *external* input and the strength of the *internal* recurrent weights. On the one hand, when $z\to 1-\sigma^{2}\approx 1$ normalization is enganged robustly and the range of stability of the circuit extends indefinitely. On the other hand, when z→0 the range of stability is narrowest.

## IV. DISCUSSION

We have established, via numerical experiments and analytical calculation, that: *(i)* the nonlinear modulation of recurrent interactions via inhibitory neurons implementing divisive normalization makes neural networks more stable than unmodulated recurrent linear models; *(ii)* the breakdown of normalization, due to substantial recurrent amplification which is not compensated by an equally strong input drive, occurs concomitantly with the onset of critical slowing down in a broad class of random neural networks.

Our results demonstrate that, at low input drives, increasing the recurrent synaptic strength turns the fixed point into a spiral attractor, as indicated by the damped oscillations in Fig. 3c and Fig. S9 (see Supplementary Section V D for details on how to determine the frequency of oscillations). Crucially, the oscillations begin at the same parameter conditions where we observe the loss of normalization and onset of critical slowing down. This suggests a strong link between these phenomena. Consequently, the detection of such recurrence-driven oscillations under a weak input drive could provide an experimental signature of a non-normalized neural circuit nearing a critical transition. For example, experimental evidence suggests that neural circuits in individuals with autism spectrum disorder exhibit both failure of normalization [43, 44] and excess variabilty (i.e., near the tipping point of instability) [45, 46]. Hence, we predict that such neural circuits will also exhibit critical slowing down, which can be measured as the elapsed time to reach steady state. Increased neural variability and noise correlations, measured across trials of same stimulus presentation, are also characteristic markers of critical slowing down (see Supplementary Section V G).

We noticed that, in the whole phase of critical slowing down, the spectrum of the Jacobian contains a large number of zero eigenvalues in the large N limit, corresponding to the emergence of multiple long time scales. Recently, it has been noted [47] that generating many long time scales in linear models requires fine tuning of the recurrent weights. In our model, many long time scales emerge for a broad range of values of the recurrent interaction strength, hence without fine tuning, suggesting that normalization (or other similar forms of multiplicative inhibitory modulation) might be the key mechanism to generate a full spectrum of slow modes in brain dynamics. A comprehensive analysis of the Jacobian’s spectrum, including the determination of the volume of zero modes, and the nature of the degenerate attractor will be presented elsewhere.

## Supplementary Material

Supplement 1

## Data Availability

No new data were generated in this work.
