# Supplementary material for: Stabilization of recurrent neural networks through divisive normalization
Source: bioRxiv. 2025 May 21:2025.05.16.654567. Preprint. [Version 1] doi: 10.1101/2025.05.16.654567 (PMC12139785; doi:10.1101/2025.05.16.654567)
Supplement: Supplement 1 [file NIHPP2025.05.16.654567v1-supplement-1.pdf]

bioRxiv preprint doi: https://doi.org/10.1101/2025.05.16.654567; this version posted May 21, 2025. The copyright holder for this preprint (which was not certified by peer review) is the author/funder, who has granted bioRxiv a license to display the preprint in perpetuity. It is made available under aCC-BY 4.0 International license.

V. SUPPLEMENTARY METHODS

A. Numerical study of ORGaNICs' fixed-point

To produce Fig. 2 in the main text we simulated an ORGaNICs network comprising $N = 100$ principal neurons with parameters set to $\sigma = 0.1$ and $\tau_y = \tau_a$. For each chosen value of the recurrent interaction strength Δ, we generated an ensemble of 10^4 recurrent connectivity matrices $W = I + K$. This was achieved by first sampling the entries of an auxiliary matrix L from a Gaussian distribution $\mathcal{N}(0, \Delta^2/N)$, and then defining the symmetric interaction matrix $K = (L + L^\top)/2$. This prescription yields a symmetric random matrix K, whose entries are normally distributed according to

$$K_{ij} = \begin{cases} \mathcal{N}\left(\frac{\mu}{N}, \frac{\Delta^2}{N}\right) \ , \ i = j \\ \mathcal{N}\left(\frac{\mu}{N}, \frac{\Delta^2}{2N}\right) \ , \ i \neq j \end{cases} \tag{9}$$

The network dynamics were simulated using the explicit Euler method (starting with a zero initial condition for all the neurons) with time step $dt = 0.05 \times \tau_y$, using a delocalized input drive $\mathbf{z}$ where each component $z_i = z/\sqrt{N}$ (ensuring $||\mathbf{z}|| = z$). We analyzed the steady-state behavior, identifying whether trajectories converged to a stable fixed point, diverged (indicating an unstable fixed point), or entered a limit cycle. For instances resulting in a stable fixed point, we computed the trial-averaged mean response $\mathbb{E}[y_i]$ and its standard deviation $\sqrt{\mathrm{Var}(y_i)}$ across the ensemble. Furthermore, we calculated the Jacobian matrix of the dynamical system at each stable fixed point using automatic differentiation [41]. Finally, as shown in Fig. 2 and Fig. S1, we plotted the mean response and its standard deviation as a function of the input drive z for different values of Δ. These plots are colored based on the average real part of the largest eigenvalue (in units of $1/\tau_y$) of the Jacobian matrix across trials, indicating the slowest mode of the dynamics near the fixed point.

bioRxiv preprint doi: https://doi.org/10.1101/2025.05.16.654567; this version posted May 21, 2025. The copyright holder for this preprint (which was not certified by peer review) is the author/funder, who has granted bioRxiv a license to display the preprint in perpetuity. It is made available under aCC-BY 4.0 International license.

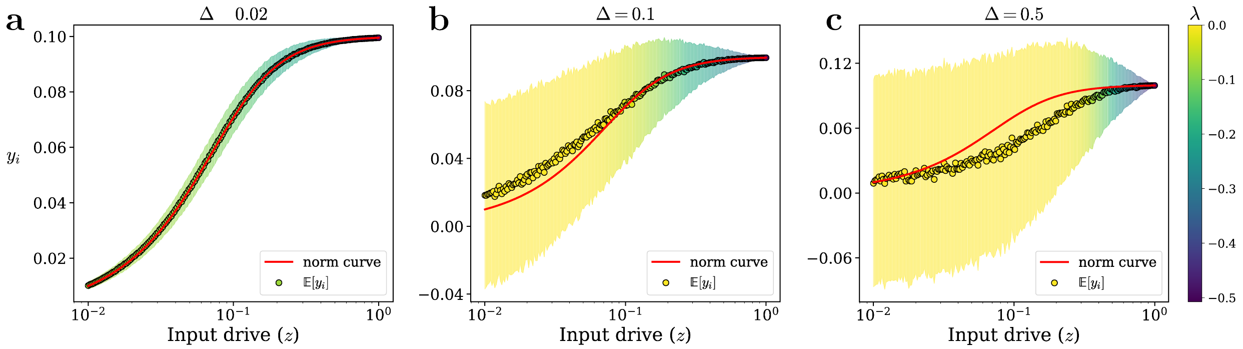

FIG. S1. **Numerical exploration of ORGaNICs' fixed-point statistics.** For each panel, we plot the fixed-point average response $\mathbb{E}[y]$ (dots) and its std. dev. (shaded area) as a function of the normalized input drive z (with $z_i = z/\sqrt{N}$, $N = 100$), for an E:I balanced recurrence matrix K of zero mean and standard deviation Δ, with the same parameters as used in Fig. 2. The solid red curve indicates the normalization equation Eq.(1). The shading color encodes the real part of the largest Jacobian eigenvalue at the fixed point, averaged over samples (always < 0 when convergence is stable). **a,** $\Delta = 0.02$: recurrent interactions are weak, yielding minimal variance around the normalization curve. **b,** $\Delta = 0.1$: moderate recurrence induces variability in the responses across random samples of the recurrent weights at small z, but the mean follows the normalization curve. **c,** $\Delta = 0.5$: strong recurrence dramatically increases the variability at small z; the mean also starts to deviate from the normalization curve.

B. Finite size analysis of the distribution $P(\lambda)$

In this section, we investigate systematically the finite size behavior of the distribution of the largest eigenvalue of the Jacobian at the fixed point $P(\lambda)$. In Fig. S2 we show $P(\lambda)$ for several values of N and Δ. At fixed Δ, we find that $P(\lambda)$ becomes sharply peaked as N increases and tends to a delta function, $P(\lambda) \to \delta(\lambda - \lambda_{gap})$ in the limit $N \to \infty$, where λ_{gap} is nonzero and negative when the circuit is stable (see Fig. S2a) and equal to zero when the circuit is critically slowed down (see Fig. S2b,c,d), i.e.

$$\begin{aligned} \lambda_{gap} < 0 \quad &\to \quad \text{stable} \ , \\ \lambda_{gap} = 0 \quad &\to \quad \text{critical slowing down} \ . \end{aligned} \tag{10}$$

bioRxiv preprint doi: https://doi.org/10.1101/2025.05.16.654567; this version posted May 21, 2025. The copyright holder for this preprint (which was not certified by peer review) is the author/funder, who has granted bioRxiv a license to display the preprint in perpetuity. It is made available under aCC-BY 4.0 International license.

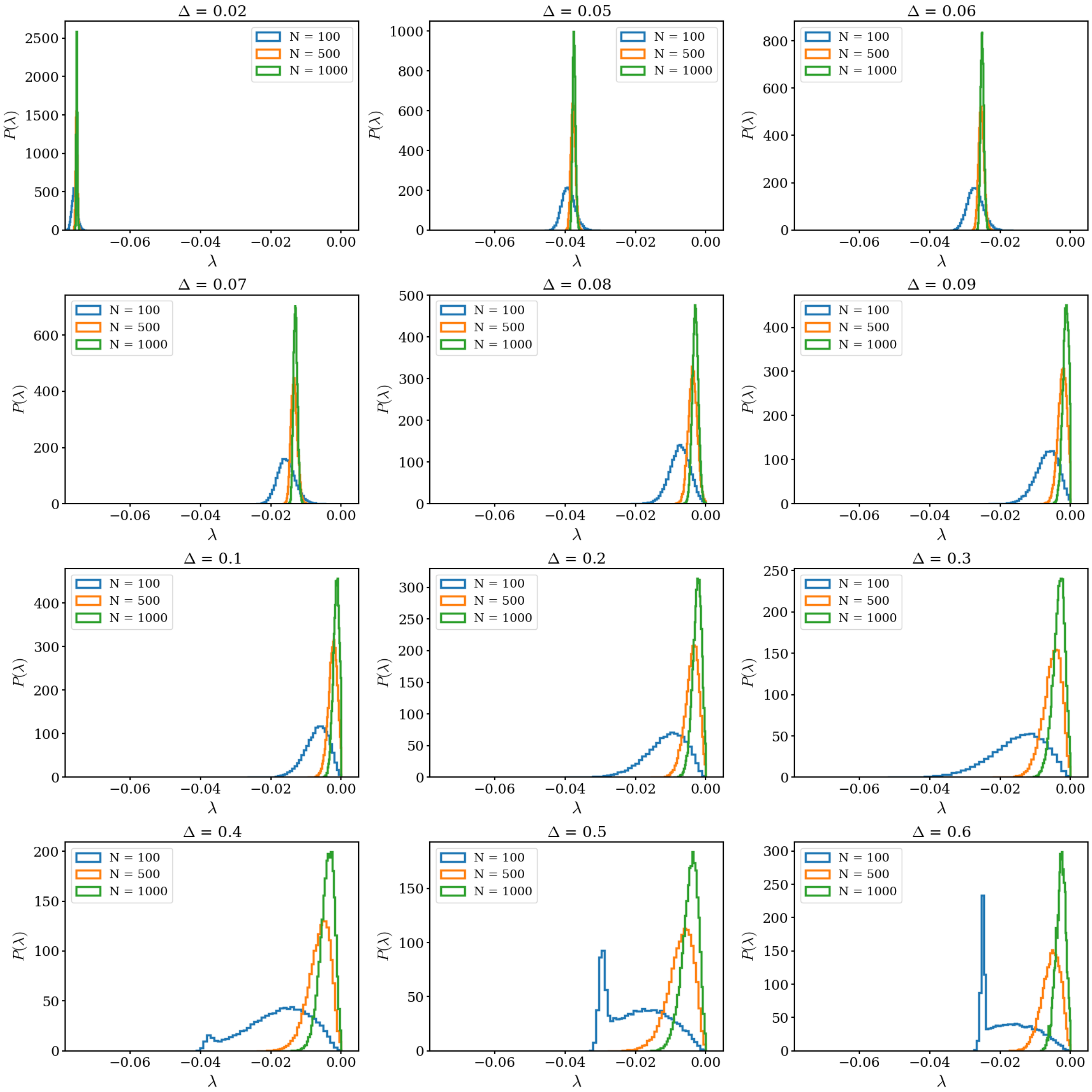

FIG. S2. **Distribution of the Jacobian's largest eigenvalue (λ) for varying system sizes (N).** Distribution of the largest eigenvalue of the Jacobian at the fixed point computed several values of Δ for different system sizes (N = 100, 500 and 1000). The input drive, simulation parameters, and the parameters of ORGaNICs are the same as those used for generating Fig. 3 in the main text. Each panel plots the distribution for different values of Δ. As system size increases, finite-size fluctuations narrow, sharpening the gap edge and more clearly revealing the approach of the rightmost eigenvalue toward zero at $\Delta \approx 0.09$. For $\Delta < \Delta_{csd}$ (panel a), all sizes exhibit a clear gap from zero; for $\Delta \geq \Delta_{csd}$ the largest-N curve touches zero most sharply.

bioRxiv preprint doi: https://doi.org/10.1101/2025.05.16.654567; this version posted May 21, 2025. The copyright holder for this preprint (which was not certified by peer review) is the author/funder, who has granted bioRxiv a license to display the preprint in perpetuity. It is made available under aCC-BY 4.0 International license.

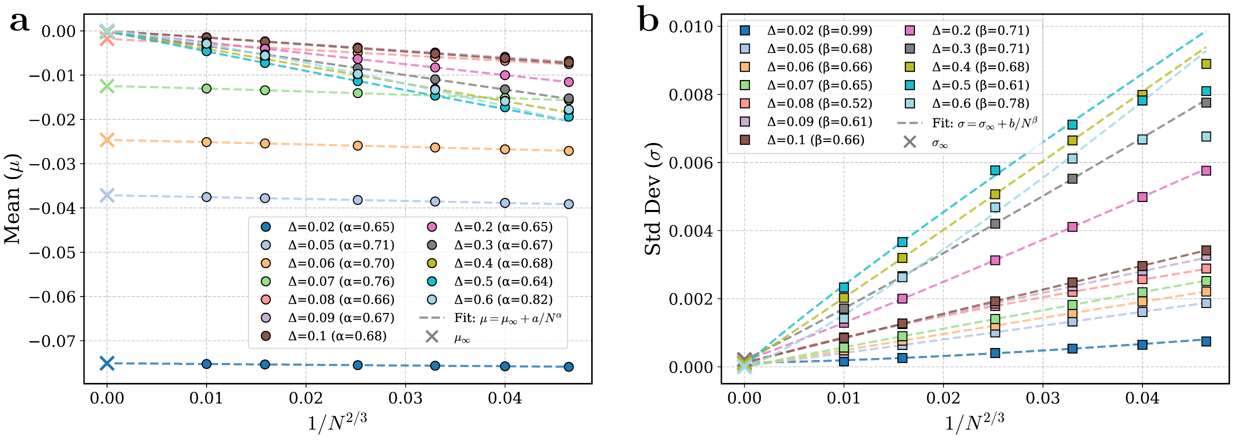

FIG. S3. **Finite size scaling analysis.** The mean and standard deviation of the largest eigenvalue of the Jacobian are shown. The input drive, simulation parameters, and the parameters of ORGaNICs are the same as those used for generating Fig. 3 in the main text. **a,** The mean μ of the largest eigenvalue λ of the Jacobian matrix at the fixed point as a function of $1/N^{2/3}$ for several values of Δ. Dashed lines represent fits following the functional form $\mu = \mu_\infty + a/N^\alpha$, where μ_∞, a, and α are fitting parameters and the fits are performed using the four largest system sizes. The 'x' markers correspond to the extrapolated mean for the infinite system size (μ_∞). The values α in the legend correspond to the fitted slopes and they are close to $2/3$ for nearly all values of Δ. μ_∞ vanishes for all values of Δ where we observe critical slowing down ($\Delta \gtrsim 0.09$). **b,** The standard deviation σ of the largest eigenvalue λ of the Jacobian matrix at the fixed point as a function of $1/N^{2/3}$ for several values of Δ. Dashed lines represent fits following the functional form $\sigma = \sigma_\infty + b/N^\beta$, where σ_∞, b, and β are fitting parameters, and fits are performed using the four largest system sizes. The fits extrapolate to a vanishing standard deviation for the infinite system size ($\sigma_\infty \approx 0$), indicating that fluctuations of λ vanish in the thermodynamic limit.

bioRxiv preprint doi: https://doi.org/10.1101/2025.05.16.654567; this version posted May 21, 2025. The copyright holder for this preprint (which was not certified by peer review) is the author/funder, who has granted bioRxiv a license to display the preprint in perpetuity. It is made available under aCC-BY 4.0 International license.

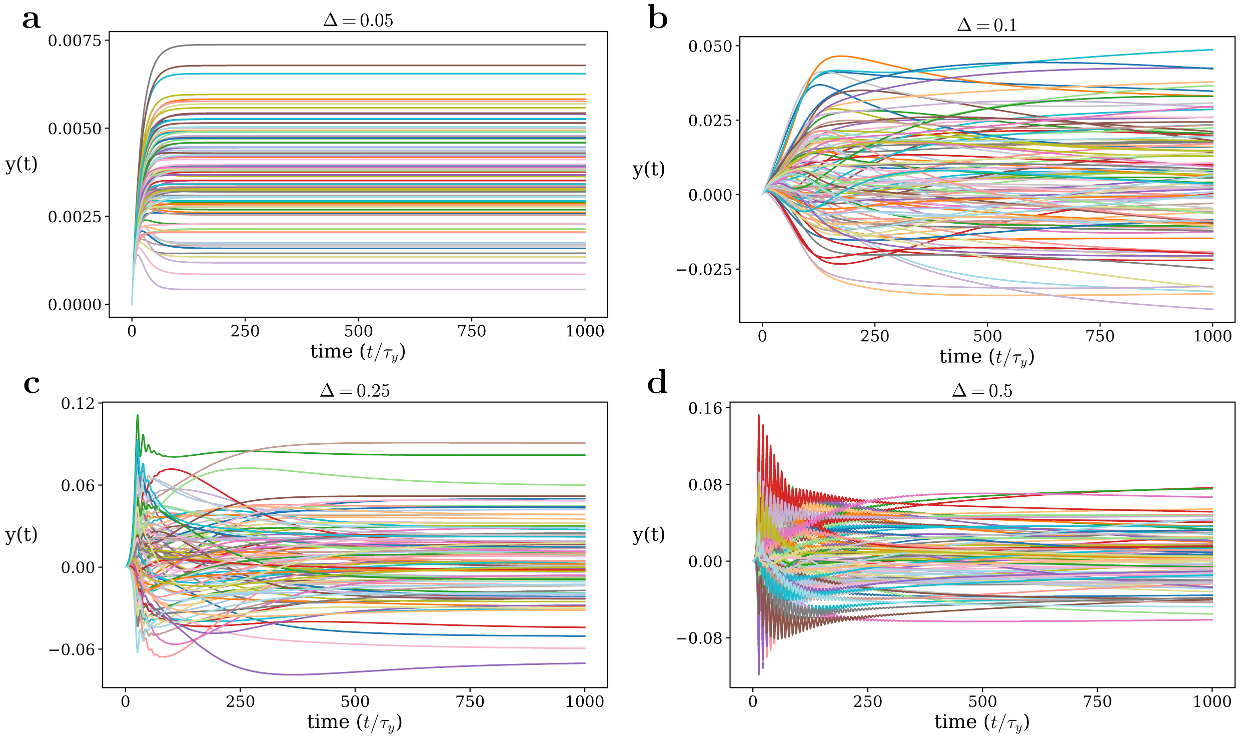

FIG. S4. **Neuronal trajectories for different recurrent synaptic strength Δ.** Each curve traces the time evolution of a distinct principal neuron's response for a given value of Δ (recurrent interaction strength). The input type and the parameters of ORGaNICs are the same as those used for generating Fig. 3 in the main text. We plot the trajectories for 100 neurons selected randomly from the 1000. In the stable regime ($\Delta = 0.05$), trajectories converge rapidly, whereas in the critical-slowing regime ($\Delta = 0.10$, 0.25, and 0.50) convergence is markedly slower.

bioRxiv preprint doi: https://doi.org/10.1101/2025.05.16.654567; this version posted May 21, 2025. The copyright holder for this preprint (which was not certified by peer review) is the author/funder, who has granted bioRxiv a license to display the preprint in perpetuity. It is made available under aCC-BY 4.0 International license.

C. Analytical calculation of the threshold for loss of normalization

Let us consider the ORGaNICs fixed point equations obtained by setting to zero the time derivative in Eq. (3) of the main text:

$$\begin{aligned} y_i &= z_i + (1-\sqrt{a})\sum_{j=1}^{N} W_{ij} y_j \ , \\ a &= \sigma^2 + a\sum_{j=1}^{N} y_j^2 \ , \end{aligned} \tag{11}$$

where we used the fact that firing rates are related to membrane potentials via

$$\begin{aligned} y_i^+ &= \lfloor y_i \rfloor^2 \ , \\ a^+ &= \sqrt{\lfloor a \rfloor} \ , \end{aligned} \tag{12}$$

and we further assumed that $a \geq 0$, which can be checked *a posteriori* to always hold true. To expand around the identity matrix we set

$$W = I + K \ , \tag{13}$$

where K is a small correction. The conditions under which the perturbation K can be considered small with respect to the identity will be deduced later on in our calculation. Inserting Eq. (13) into Eq. (11) we obtain

$$\begin{aligned} \sqrt{a} y_i &= z_i + (1-\sqrt{a})\sum_{j=1}^{N} K_{ij} y_j \ , \\ a &= \frac{\sigma^2}{1-||\mathbf{y}||^2} \ , \end{aligned} \tag{14}$$

where we defined the squared norm as $||\mathbf{y}||^2 = \sum_{j=1}^{N} y_j^2$. We look for a solution to Eq. (14) in the form of a series

$$\begin{aligned} y_i &= y_i^{(0)} + y_i^{(1)} + y_i^{(2)} + \cdots \ , \\ a &= a^{(0)} + a^{(1)} + a^{(2)} + \cdots \ , \end{aligned} \tag{15}$$

where $y_i^{(1)}, a^{(1)}$ are of the same order of magnitude of the perturbation K, the quantities $y_i^{(2)}, a^{(2)}$ are of second order, and so on. To find the first approximation, we substitute $y_i = y_i^{(0)} + y_i^{(1)}$ and $a = a^{(0)} + a^{(1)}$ in Eq. (14) and we keep only terms up to the first order, thus obtaining

$$\begin{aligned} y_i^{(0)} + y_i^{(1)} &= z_i + \left(1-\sqrt{a^{(0)}}\right) y_i^{(0)} + \left(1-\sqrt{a^{(0)}}\right)\left(y_i^{(1)} + \sum_j K_{ij} y_j^{(0)}\right) - \frac{y_i^{(0)} a^{(1)}}{2\sqrt{a^{(0)}}} \ , \\ a^{(0)} + a^{(1)} &= \frac{\sigma^2}{1-||\mathbf{y}^{(0)}||^2} + 2\sigma^2 \frac{\mathbf{y}^{(0)}\cdot\mathbf{y}^{(1)}}{\left(1-||\mathbf{y}^{(0)}||^2\right)^2} \ , \end{aligned} \tag{16}$$

bioRxiv preprint doi: https://doi.org/10.1101/2025.05.16.654567; this version posted May 21, 2025. The copyright holder for this preprint (which was not certified by peer review) is the author/funder, who has granted bioRxiv a license to display the preprint in perpetuity. It is made available under aCC-BY 4.0 International license.

where $\mathbf{y}^{(0)} \cdot \mathbf{y}^{(1)} = \sum_i y_i^{(0)} y_i^{(1)}$ is the usual dot product. Equating the terms of order zero on both sides of Eq. (16) we obtain

$$\begin{aligned} y_i^{(0)} &= \frac{z_i}{\sqrt{a^{(0)}}} \;, \\ a^{(0)} &= \frac{\sigma^2}{1 - ||\mathbf{y}^{(0)}||^2} \;, \end{aligned} \tag{17}$$

which, as it should, is equivalent to the normalization equation

$$\begin{aligned} y_i^{(0)} &= \frac{z_i}{\sqrt{\sigma^2 + ||\mathbf{z}||^2}} \;, \\ a^{(0)} &= \sigma^2 + ||\mathbf{z}||^2 \;. \end{aligned} \tag{18}$$

To find the first order corrections $y_i^{(1)}$ and $a^{(1)}$ we equate the terms of order one on both sides of Eq. (16) and we get

$$\begin{aligned} y_i^{(1)} \sqrt{a^{(0)}} &= \left(1 - \sqrt{a^{(0)}}\right) \sum_j K_{ij} y_j^{(0)} - \frac{y_i^{(0)} a^{(1)}}{2\sqrt{a^{(0)}}} \;, \\ a^{(1)} &= 2a^{(0)} \frac{\mathbf{y}^{(0)} \cdot \mathbf{y}^{(1)}}{1 - ||\mathbf{y}^{(0)}||^2} \;, \end{aligned} \tag{19}$$

where in the equation for $a^{(1)}$ we have used the definition of $a^{(0)}$ given in Eq. (17). To solve Eq. (19) we multiply the first equation by $y_i^{(0)}$ and, after summing over i, we find

$$\mathbf{y}^{(0)} \cdot \mathbf{y}^{(1)} = \frac{\sigma^2 \left(1 - \sqrt{\sigma^2 + ||\mathbf{z}||^2}\right)}{\left(\sigma^2 + ||\mathbf{z}||^2\right)^{5/2}} \mathbf{z}^\top K \mathbf{z} \;, \tag{20}$$

from which we can compute $a^{(1)}$. Substituting this result into Eq. (19) we can express $y_i^{(1)}$ as a function of z and K as

$$\boxed{\begin{aligned} y_i^{(1)} &= G(||\mathbf{z}||) \left(\sum_j K_{ij} z_j - z_i \frac{\mathbf{z}^\top K \mathbf{z}}{\sigma^2 + ||\mathbf{z}||^2} \right) \;, \\ G(||\mathbf{z}||) &= \frac{1 - \sqrt{\sigma^2 + ||\mathbf{z}||^2}}{\sigma^2 + ||\mathbf{z}||^2} \end{aligned}} \tag{21}$$

Having found the general form of the first order correction, we move next to consider the case of a random matrix K sampled from the so-called Gaussian Orthogonal Ensemble (GOE).

We consider the ensemble of symmetric random matrices K, whose entries are normally distributed according to

$$K_{ij} = \begin{cases} \mathcal{N}\left(\frac{\mu}{N}, \frac{\Delta^2}{N}\right) \;, & i = j \\ \mathcal{N}\left(\frac{\mu}{N}, \frac{\Delta^2}{2N}\right) \;, & i \neq j \end{cases} \tag{22}$$

bioRxiv preprint doi: https://doi.org/10.1101/2025.05.16.654567; this version posted May 21, 2025. The copyright holder for this preprint (which was not certified by peer review) is the author/funder, who has granted bioRxiv a license to display the preprint in perpetuity. It is made available under aCC-BY 4.0 International license.

We can compute the average of $y_i^{(1)}$ in Eq.(21) straightforwardly and find

$$\mathbb{E}\big[y_i^{(1)}\big] = \frac{\mu}{N} G(||\mathbf{z}||) \left[\sum_j z_j - \frac{z_i}{\sigma^2 + ||\mathbf{z}||^2} \Big(\sum_j z_j \Big)^2 \right] . \tag{23}$$

A little bit of algebra yields the following expression for the second moment

$$\begin{aligned} \mathbb{E}\Big[\big(y_i^{(1)}\big)^2\Big] &= G^2(||\mathbf{z}||) \left[Q_i - \frac{2z_i}{\sigma^2 + ||\mathbf{z}||^2} P_i + \frac{z_i^2}{\big(\sigma^2 + ||\mathbf{z}||^2\big)^2} R \right] , \\ Q_i &= \frac{\Delta^2}{2N} \Big(z_i^2 + ||\mathbf{z}||^2 \Big) - \frac{\mu^2}{N^2} \left[||\mathbf{z}||^2 - \Big(\sum_j z_j \Big)^2 \right] , \\ P_i &= \frac{\Delta^2}{N} z_i ||\mathbf{z}||^2 - \frac{\mu^2}{N^2} \left[2 z_i ||\mathbf{z}||^2 - z_i^3 - \Big(\sum_j z_j \Big)^3 \right] , \\ R &= \frac{\Delta^2}{N} ||\mathbf{z}||^4 - \frac{\mu^2}{N^2} \left[2||\mathbf{z}||^4 - \Big(\sum_j z_j^4 \Big) - \Big(\sum_j z_j \Big)^4 \right] . \end{aligned} \tag{24}$$

Having found the general expressions for the first and second moments of $y_i^{(1)}$, next we discuss the case $\mu = 0$ (E:I balance), corresponding to having an equal number (on average) of positive and negative synaptic weights. Mathematically, this is obtained by setting to zero the mean ($\mu = 0$) of the random matrix entries K_{ij}. The mean and variance of the perturbation $y_i^{(1)}$ simplify considerably and read

$$\begin{aligned} \mathbb{E}\big[y_i^{(1)}\big] &= 0 , \\ \mathbb{E}\Big[\big(y_i^{(1)}\big)^2\Big] &= G^2(||\mathbf{z}||) \left[\frac{\Delta^2}{2N} \big(z_i^2 + ||\mathbf{z}||^2 \big) - \frac{\Delta^2}{N} \frac{2 z_i^2 ||\mathbf{z}||^2}{\sigma^2 + ||\mathbf{z}||^2} + \frac{\Delta^2}{N} \frac{z_i^2 ||\mathbf{z}||^4}{\big(\sigma^2 + ||\mathbf{z}||^2\big)^2} \right] . \end{aligned} \tag{25}$$

In the following, we will consider two types of input drives, a **delocalized** input drive, characterized by a vector $\mathbf{z}$ with all entries z_i equal to

$$z_i = \frac{z}{\sqrt{N}} \qquad \text{delocalized input drive} , \tag{26}$$

and the case of a **localized** input drive where all entries are equal to zero but one, for example z_1, and denoted

$$z_i = z \delta_{i1} \qquad \text{localized input drive} . \tag{27}$$

1. Delocalized input drive

When the input drive is delocalized, the variance of the perturbation becomes

$$\mathbb{E}\Big[\big(y_i^{(1)}\big)^2\Big] = \frac{\Delta^2 z^2}{2N} G(||\mathbf{z}||)^2 + O(N^{-2}) . \tag{28}$$

bioRxiv preprint doi: https://doi.org/10.1101/2025.05.16.654567; this version posted May 21, 2025. The copyright holder for this preprint (which was not certified by peer review) is the author/funder, who has granted bioRxiv a license to display the preprint in perpetuity. It is made available under aCC-BY 4.0 International license.

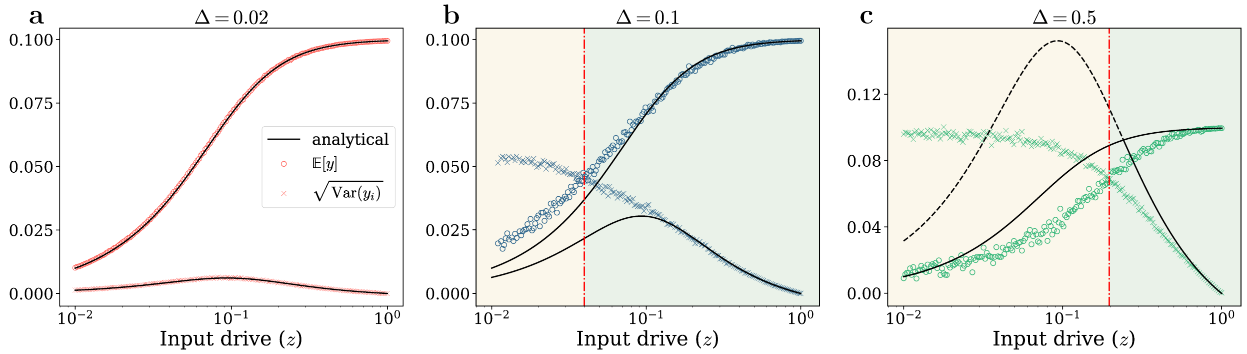

FIG. S5. **Loss of normalization.** Following the analysis presented in Fig. 4, this figure illustrates the mean (circles) and standard deviation (crosses) of the fixed point neural responses versus the input norm z for three additional values of the recurrent interaction strength: $\Delta = 0.02$, $\Delta = 0.1$, and $\Delta = 0.5$. The network consists of $N = 100$ neurons, and each point is found using 1000 realizations. **a,** $\Delta = 0.02$, the std. dev. remains below the mean across all input drives, indicating preserved normalization. **b,** $\Delta = 0.1$ and **c,** $\Delta = 0.5$, the std. dev. exceeds the mean at small z, demonstrating loss of normalization, defined by the crossing point (dashed red line). The theoretical predictions from perturbation theory (black curves) match the numerical simulations well in the normalized regime (mean > std. dev.). Discrepancies increase at small z for larger Δ, where normalization is lost.

The threshold $\Delta_{loss}(z)$ separating the phase where responses are normalized from the phase where they are not is obtained by equating the mean of the response $\mathbb{E}[y_i]$ to its standard deviation $\sqrt{\mathrm{Var}[y_i]}$, yielding

$$1 = \frac{\mathbb{E}[y_i]}{\sqrt{\mathrm{Var}[y_i]}} = \frac{y_i^{(0)}}{\sqrt{\mathbb{E}\left[\left(y_i^{(1)}\right)^2\right]}} \quad \to \quad \frac{z}{\sqrt{N}}\frac{1}{\sqrt{\sigma^2+z^2}} = \frac{\Delta z}{\sqrt{2N}} G(||\mathbf{z}||) \ , \tag{29}$$

from which we obtain

$$\frac{\Delta_{loss}(z)}{\sqrt{2}} = \frac{\sqrt{\sigma^2+z^2}}{1-\sqrt{\sigma^2+z^2}} \ , \tag{30}$$

which is Eq. (8) in the main text. In Fig. S5 we compare the analytical approximations for the mean and variance of the responses with the exact numerical values. The agreement is excellent at small Δ, since the neural responses are always normalized, i.e. normalization always holds. For larger Δ the analytical and numerical results also agree well at large input drive, where the responses are normalized. At small z, normalization breaks down as well as the analytical approximation.

bioRxiv preprint doi: https://doi.org/10.1101/2025.05.16.654567; this version posted May 21, 2025. The copyright holder for this preprint (which was not certified by peer review) is the author/funder, who has granted bioRxiv a license to display the preprint in perpetuity. It is made available under aCC-BY 4.0 International license.

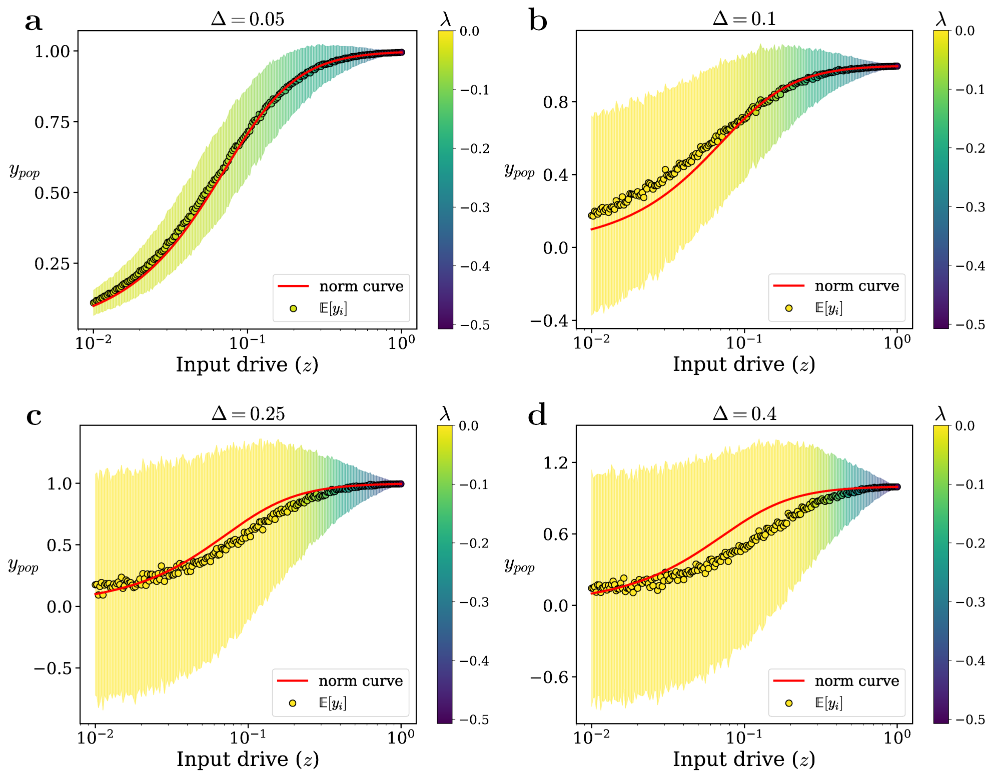

FIG. S6. **Numerical solution of the ORGaNICs' fixed point equations for** **localized input drive**. **a,** Fixed-point average response $\mathbb{E}[y_{pop}]$, defined in Eq. (32), as a function of the input drive z (chosen as $z_i = z\delta_{1i}$) for an E:I balanced recurrence matrix K with zero mean and recurrent interaction strength $\Delta = 0.05$ and $N = 100$ neurons, obtained by solving numerically Eq. (3) using the explicit Euler method with time step $dt = 0.05 \times \tau_y$. The semisaturation constant is $\sigma = 0.1$ and the time constants $\tau_y = \tau_a$. Each point is an average over 1000 realizations of the synaptic weights K_{ij}. The neural responses still follow, on average, the normalization Eq. (1) (solid red curve), but pick up a variance in presence of recurrent connections, represented by the shaded area around the data points. The color code of the shaded area represents the real part of the largest eigenvalue of the Jacobian at the fixed point averaged over samples, whose value is well below 0 for all z. **b, c, d** For $\Delta = 0.1, 0.25, 0.4$ the average response is still normalized, but the variance is bigger than in (**a**). For sufficiently small input drives, the largest eigenvalue of the Jacobian at the fixed point vanishes and, as a consequence, convergence to the fixed point occurs on long time scales, a phenomenon known as critical slowing down.

2. *Localized input drive*

In this section, we show that our results and conclusions are the same for the localized input. We consider the extreme case where $\mathbf{z}$ is a one-hot vector with

$$z_i = z\delta_{i1} \ . \tag{31}$$

bioRxiv preprint doi: https://doi.org/10.1101/2025.05.16.654567; this version posted May 21, 2025. The copyright holder for this preprint (which was not certified by peer review) is the author/funder, who has granted bioRxiv a license to display the preprint in perpetuity. It is made available under aCC-BY 4.0 International license.

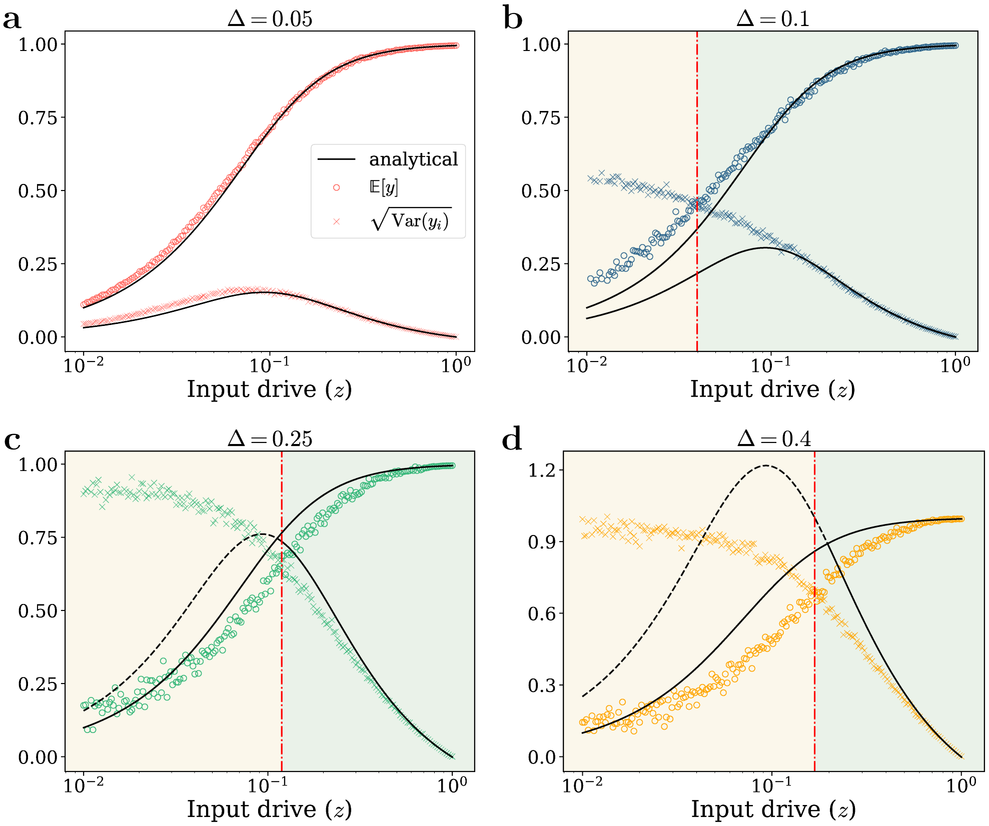

FIG. S7. **Loss of normalization for localized input drive.** **a,** Mean (circles) and standard deviation (crosses) of the fixed point response y_{pop}, defined in Eq. (32), as a function of the norm z of the localized input drive $z_i = z\delta_{1i}$ for an E:I balanced recurrence matrix K with zero mean and recurrent interaction strength $\Delta = 0.05$. We used $N = 100$ neurons and averaged over 10^3 realizations of the random matrix K. The analytical approximations (solid curves) for the mean Eq. (33) and standard deviation Eq. (34) of the response, computed with perturbation theory, show a good agreement between the theoretical and numerical solutions. In this case the standard deviation is smaller than the mean for all values of z, so the neural responses are always normalized. **b, c, d** Same as in **a**, but using $\Delta = 0.1, 0.25, 0.4$. The standard deviation is smaller than the mean at large input drive, but gets bigger than the mean at small input drive. The value of z where the two curves cross each other, given by Eq. (37), defines the point at which the neural responses lose normalization. The analytical approximations are in good agreement with numerical simulations for almost all values of the input drive (notice the log scale on the abscissa), but become less accurate at small z where the responses are non-normalized.

Since the fixed point y_i depends on i we consider the sum over all responses y_{pop} defined as

$$y_{pop} = \sum_{i=1}^{N} y_i \approx \sum_{i=1}^{N} y_i^{(0)} + y_i^{(1)} \ . \tag{32}$$

The mean of y_{pop} is simply

$$\mathbb{E}[y_{pop}] = \frac{z}{\sqrt{\sigma^2 + z^2}} \ . \tag{33}$$

bioRxiv preprint doi: https://doi.org/10.1101/2025.05.16.654567; this version posted May 21, 2025. The copyright holder for this preprint (which was not certified by peer review) is the author/funder, who has granted bioRxiv a license to display the preprint in perpetuity. It is made available under aCC-BY 4.0 International license.

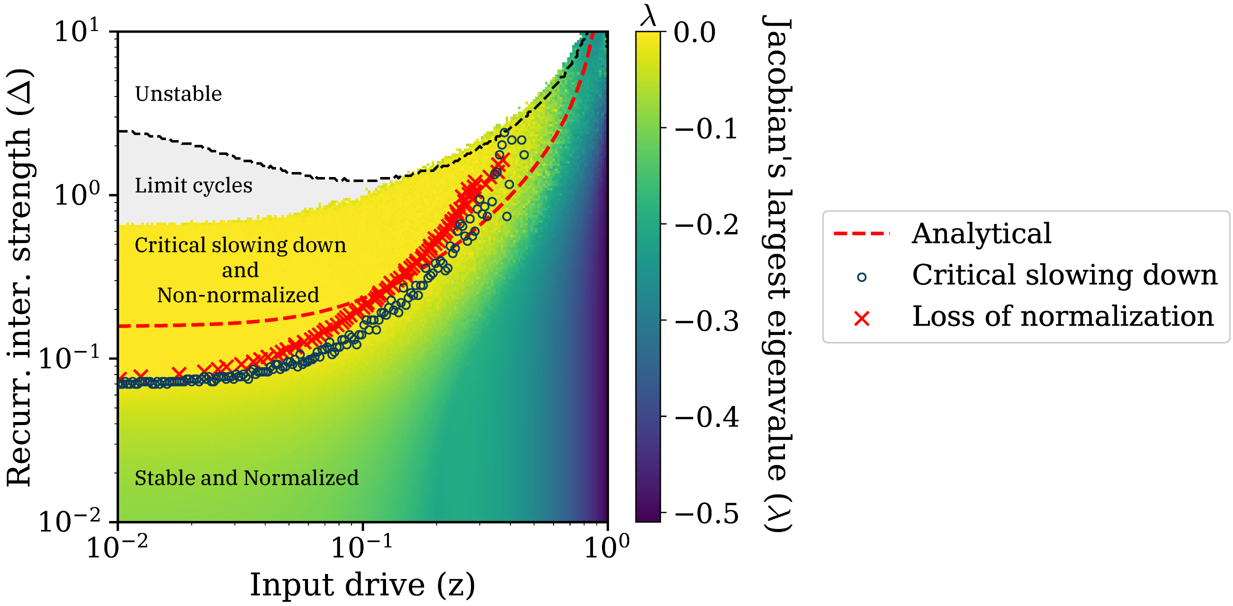

FIG. S8. **Loss of normalization predicts the onset of critical slowing down for localized input drive.** Real part of the largest eigenvalue of the Jacobian at the fixed point in the (z, Δ) plane obtained by solving numerically Eqs. (3) with a localized input drive, i.e., $z_i = z\delta_{i1}$. The parameters of ORGaNICs are the same as those used for generating Fig. 5 in the main text. Color represents the maximum value of λ across 100 trials. Circuits with small Δ are stable at any value of the input drive z and converge quickly to their fixed point, as indicated by a strictly negative eigenvalue $\lambda < 0$. Conversely, for $\Delta_{csd} < \Delta < \Delta_c$, the circuits exhibit critical slowing down, in that they approach the fixed point very slowly, as indicated by the zero eigenvalue, $\lambda = 0$. The onset of critical slowing down is defined by the first time the eigenvalue becomes zero, here denoted by the blue empty circle. The onset of slowing down is equally well captured by the red crosses, representing the boundary between the normalized and non-normalized phases. Loss of normalization is a good proxy for critical slowing down even for localized input drives. For sufficiently large Δ the circuits exhibit limit cycles and for even larger Δ they eventually become unstable, where instability is defined as trajectories diverging in at least 50% of trials.

The variance is given by

$$\mathrm{Var}[y_{pop}] = \mathbb{E}\big[\big(y_1^{(1)}\big)^2\big] + 2\sum_{i\neq 1}\mathbb{E}\big[y_1^{(1)}y_i^{(1)}\big] + \sum_{i,j\neq 1}\mathbb{E}\big[y_i^{(1)}y_j^{(1)}\big] \ . \tag{34}$$

The calculation of the expectation values gives

$$\begin{aligned}
\mathbb{E}\big[\big(y_1^{(1)}\big)^2\big] &= \frac{\Delta^2 z^2}{N} G^2(||\mathbf{z}||)\left(1 - \frac{z^2}{\sigma^2 + z^2}\right)^2 + O(N^{-2}) \ , \\
\mathbb{E}\big[y_1^{(1)}y_i^{(1)}\big] &= 0 \quad \text{for } i \neq 1 \ , \\
\mathbb{E}\big[y_i^{(1)}y_j^{(1)}\big] &= \begin{cases} \frac{\Delta^2 z^2}{2N} G^2(||\mathbf{z}||) + O(N^{-2}) & \text{for } i,j \neq 1 \\ 0 & \text{for } i = 1 \text{ or } j = 1 \ . \end{cases}
\end{aligned} \tag{35}$$

bioRxiv preprint doi: https://doi.org/10.1101/2025.05.16.654567; this version posted May 21, 2025. The copyright holder for this preprint (which was not certified by peer review) is the author/funder, who has granted bioRxiv a license to display the preprint in perpetuity. It is made available under aCC-BY 4.0 International license.

Inserting the previous expressions in Eq. (34) and keeping only the leading order in N we find

$$\mathrm{Var}[y_{pop}] = \frac{\Delta^2 z^2}{2} G^2(||\mathbf{z}||) \ . \tag{36}$$

Equating the mean and standard deviation of y_{pop} we find the $\Delta_{loss}(z)$ as

$$\frac{\mathbb{E}[y_{pop}]}{\sqrt{\mathrm{Var}[y_{pop}]}} = 1 \ \rightarrow \ \frac{\Delta_{loss}(z)}{\sqrt{2}} = \frac{\sqrt{\sigma^2 + z^2}}{1 - \sqrt{\sigma^2 + z^2}} \ , \tag{37}$$

which is the same expression as in Eq. (30).

D. Frequency of oscillations

To understand how the interplay between external stimuli and recurrent drive affects the dynamics of ORGaNICs, we investigated the system's propensity to oscillate under varying conditions. Specifically, we explored the influence of the overall input drive z and the recurrent interaction strength Δ. We systematically varied these two parameters and computed the average oscillation frequency of the network activity as the mean imaginary part of the Jacobian eigenvalues $\mathrm{Im}(\lambda_{\mathbf{J}})/(2\pi)$ evaluated at the system's fixed point. Oscillatory dynamics (spiralling fixed points) are indicated by complex eigenvalues. Fig. S9 shows the resulting heat map in the (z, Δ) plane, where we plot the mean oscillation frequency for a network of $N = 100$ neurons with time constants $\tau_y = \tau_a = 2$ msec, considering both delocalized (Fig. S9a) and localized input drives (Fig. S9b).

We find distinct dynamical regimes. When the input drive and the synaptic strength are weak, i.e., $z \lesssim 0.1$ and $\Delta \lesssim 0.1$, the circuits settle into a stable, non-oscillating fixed point. However, as the input drive increases ($z \gtrsim 0.1$), the fixed point becomes a spiral attractor, leading to oscillations falling within the gamma frequency range (30-100 Hz). Within this regime, the frequency of these input-driven oscillations scales positively with the input drive z (Fig. S9d). A different scenario unfolds when the recurrent synaptic strength is increased (Fig. S9c). For low input drive ($z \lesssim 0.1$), damped oscillations emerge beyond $\Delta \approx 0.1$. In this recurrence-driven regime, the oscillation frequency increases monotonically with Δ from 0 Hz to 80 Hz, before the attractor ultimately turns into limit cycles at higher Δ. These findings highlight the dual roles of external input and internal recurrent drive in shaping the frequency of the oscillatory behavior in ORGaNICs.

bioRxiv preprint doi: https://doi.org/10.1101/2025.05.16.654567; this version posted May 21, 2025. The copyright holder for this preprint (which was not certified by peer review) is the author/funder, who has granted bioRxiv a license to display the preprint in perpetuity. It is made available under aCC-BY 4.0 International license.

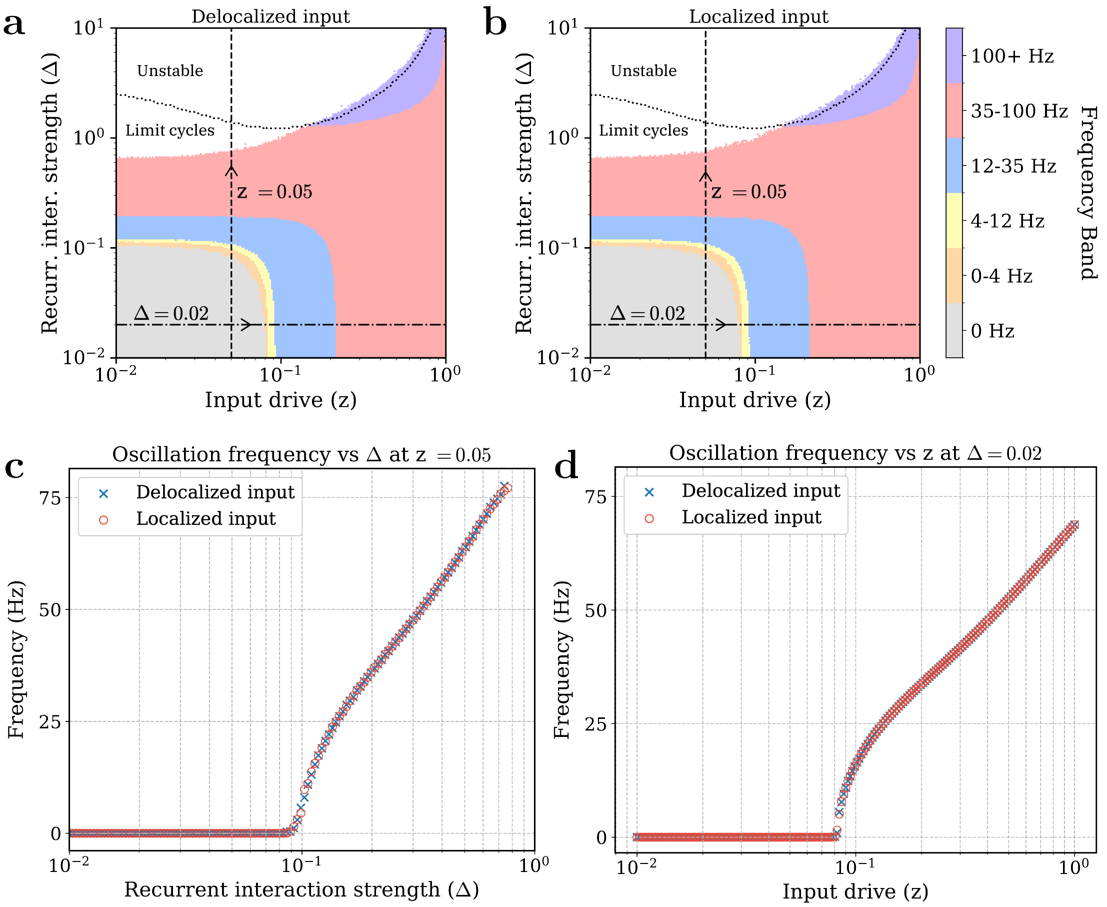

FIG. S9. **Phase diagram and oscillation frequencies in ORGaNICs.** **a**, **b**, Phase diagrams depicting the average oscillation frequency for ORGaNICs as a function of input drive (z) and recurrent interaction strength (Δ). Frequency is color-coded according to standard bands (see color bar: 0 Hz, 0-4 Hz, 4-12 Hz, 12-35 Hz, 35-100 Hz, 100+ Hz), calculated as the mean imaginary part of the Jacobian eigenvalues $\text{Im}(\lambda_{\mathbf{J}})/(2\pi)$ across trials. Results are shown for delocalized (**a**) and localized (**b**) inputs in a system with $N = 100$ neurons, semisaturation constant $\sigma = 0.1$, and time constants $\tau_y = \tau_a = 2$ msec. Dotted curves indicate the transition from limit cycles to an unstable regime, where instability is defined as trajectories diverging in at least 50% of trials. **c**, Oscillation frequency vs recurrent interaction strength (Δ) at a fixed input drive $z = 0.05$. **d**, Oscillation frequency vs input drive (z) at a fixed recurrent interaction strength $\Delta = 0.02$. Plots **c** and **d** compare delocalized (blue circles) and localized (red circles) inputs, showing minimal difference between the two input types.

E. ORGaNICs with alternative activation functions

We consider the effect of changing the activation function, which determines the firing rates (y^+) from the membrane potentials (y) of the principal neurons. In the model studied in the main text we use a quadratic activation function $y^+ = y^2$ (see phase diagram of stability in Fig. S10a). Here, we investigate two alternative models incorporating rectification in the activation function,

bioRxiv preprint doi: https://doi.org/10.1101/2025.05.16.654567; this version posted May 21, 2025. The copyright holder for this preprint (which was not certified by peer review) is the author/funder, who has granted bioRxiv a license to display the preprint in perpetuity. It is made available under aCC-BY 4.0 International license.

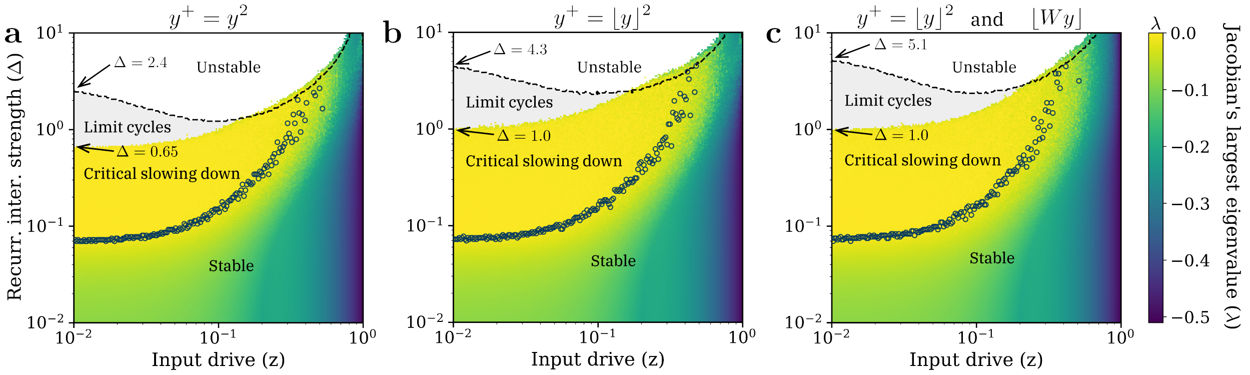

FIG. S10. **Effect of rectification on network stability.** Phase diagram in the parameter space of input drive z and recurrent interaction strength Δ (mesh size 200×200). Color represents the maximum value (across 100 trials) of the Jacobian's eigenvalue with the largest real part (λ). The input type and the parameters of ORGaNICs are the same as those used for generating Fig. 5 in the main text. The panels correspond to: **a**, Model with quadratic activation ($y^+ = y^2$), identical to the phase diagram in Fig. 5. **b**, Model with rectification in the activation function ($y^+ = \lfloor y \rfloor^2$, Eq. 38). **c**, Model with rectification applied in both the activation function and after the recurrent summation ($y^+ = \lfloor y \rfloor^2$ and $\lfloor Wy \rfloor$ term, Eq. 39). We observe that the boundaries marking the transition from critical slowing down to limit cycles, and from limit cycles to unstable dynamics (indicated by dashed curves), shift upwards in going from **a** to **b** to **c**.

defined as $\lfloor x \rfloor = \max(0, x)$, a common choice known as ReLU in artificial neural networks.

First, we introduce rectification such that the firing rate is calculated as $y^+ = \lfloor y \rfloor^2$. This gives us the following dynamical system (see phase diagram in Fig. S10b):

$$\begin{cases} \tau_y \dot{y}_i = -y_i + z_i + (1 - a^+) \sum_{j=1}^{N} W_{ij} \lfloor y \rfloor_i \\ \tau_a \dot{a} = -a + \sigma^2 + \left(\sum_{i=1}^{N} y_i^+ \right) a \ , \end{cases} \tag{38}$$

In the second model, we explore a different placement for rectification. While still using the rectified firing rate $y^+ = \lfloor y \rfloor^2$, we apply rectification after the weighted recurrent inputs have been summed, in the dynamical equation for y. This leads to the following dynamical system (see corresponding phase diagram in Fig. S10c):

$$\begin{cases} \tau_y \dot{y}_i = -y_i + z_i + (1 - a^+) \left\lfloor \sum_{j=1}^{N} W_{ij} y_i \right\rfloor \\ \tau_a \dot{a} = -a + \sigma^2 + \left(\sum_{i=1}^{N} y_i^+ \right) a \ , \end{cases} \tag{39}$$

bioRxiv preprint doi: https://doi.org/10.1101/2025.05.16.654567; this version posted May 21, 2025. The copyright holder for this preprint (which was not certified by peer review) is the author/funder, who has granted bioRxiv a license to display the preprint in perpetuity. It is made available under aCC-BY 4.0 International license.

The model in Eq. (39) is not neurobiologically relevant, but is relevant for designing ML architectures [18].

We analyzed the stability of these models by examining their phase diagrams in the parameter space of input drive (z) and recurrent interaction strength (Δ), shown in Fig. S10. We find that introducing these alternative forms of rectification do not qualitatively change the network's phase diagram. However, the boundaries that mark the transition from critical slowing down to limit cycles, and from limit cycles to unstable dynamics, shift towards larger values of Δ, when going from panel (a) to (b) to (c) of Fig. S10. Therefore, introducing rectification increases the range of parameters for which the neuron's trajectories remain bounded (including the limit cycles regime).

F. E-I imbalanced recurrent networks

In this section, we investigate the impact of excitation-inhibition (E-I) imbalance in the recurrent weight matrix W on the stability of ORGaNICs. We introduce E-I imbalance by setting a non-zero mean μ for the entries of the recurrent connectivity matrix K, such that $K_{ii} \sim \mathcal{N}(\mu/N, \Delta^2/N)$ and $K_{ij} \sim \mathcal{N}(\mu/N, \Delta^2/2N)$ for $i \neq j$. This introduces net inhibition (for $\mu < 0$) or net excitation ($\mu > 0$) in K. We generated phase diagrams, shown in Fig. S11, analogous to Fig. 5 for different values of $\mu = [0.0,\, 0.05,\, 0.1,\, 0.25,\, 0.5,\, 1.0,\, -0.1,\, -0.5,\, -1.0]$, using a delocalized input drive $z_i = z/\sqrt{N}$ and network parameters $N = 100$, $\sigma = 0.1$, $\tau_y = \tau_a$. We observe three main things:

1. increasing excitation (larger positive μ) shifts the onset of critical slowing down towards larger values of the recurrent interaction strength Δ. For strong excitatory imbalance (e.g., $\mu = 1.0$), the network transitions from the stable regime to the limit cycle regime at small input drive z without undergoing critical slowing down;

2. increasing inhibition (larger negative μ) makes the circuit operate in the critically slowed down regime for larger values of Δ at any input drive z. For strong inhibitory imbalance (e.g., $\mu = -1.0$), the circuit remains stable across all z without entering into limit cycles at small z;

3. most importantly, the loss of normalization is still a good predictor of the onset of critical slowing down across all values of μ.

We also examined how E-I imbalance affects the oscillation frequencies in ORGaNICs (Fig. S12). Upon increasing excitation ($\mu > 0$), the region exhibiting high-frequency oscillations (gamma band

bioRxiv preprint doi: https://doi.org/10.1101/2025.05.16.654567; this version posted May 21, 2025. The copyright holder for this preprint (which was not certified by peer review) is the author/funder, who has granted bioRxiv a license to display the preprint in perpetuity. It is made available under aCC-BY 4.0 International license.

and higher) expands. For strong excitation (e.g., $\mu = 1.0$), the network tends to oscillate at high frequencies across a wider range of Δ and z values. This suggests that net excitation in the recurrent connections promotes faster oscillations. On the contrary, increasing inhibition (Fig. S12g,h,i) ($\mu < 0$) promotes slower oscillations, especially at large input drives.

bioRxiv preprint doi: https://doi.org/10.1101/2025.05.16.654567; this version posted May 21, 2025. The copyright holder for this preprint (which was not certified by peer review) is the author/funder, who has granted bioRxiv a license to display the preprint in perpetuity. It is made available under aCC-BY 4.0 International license.

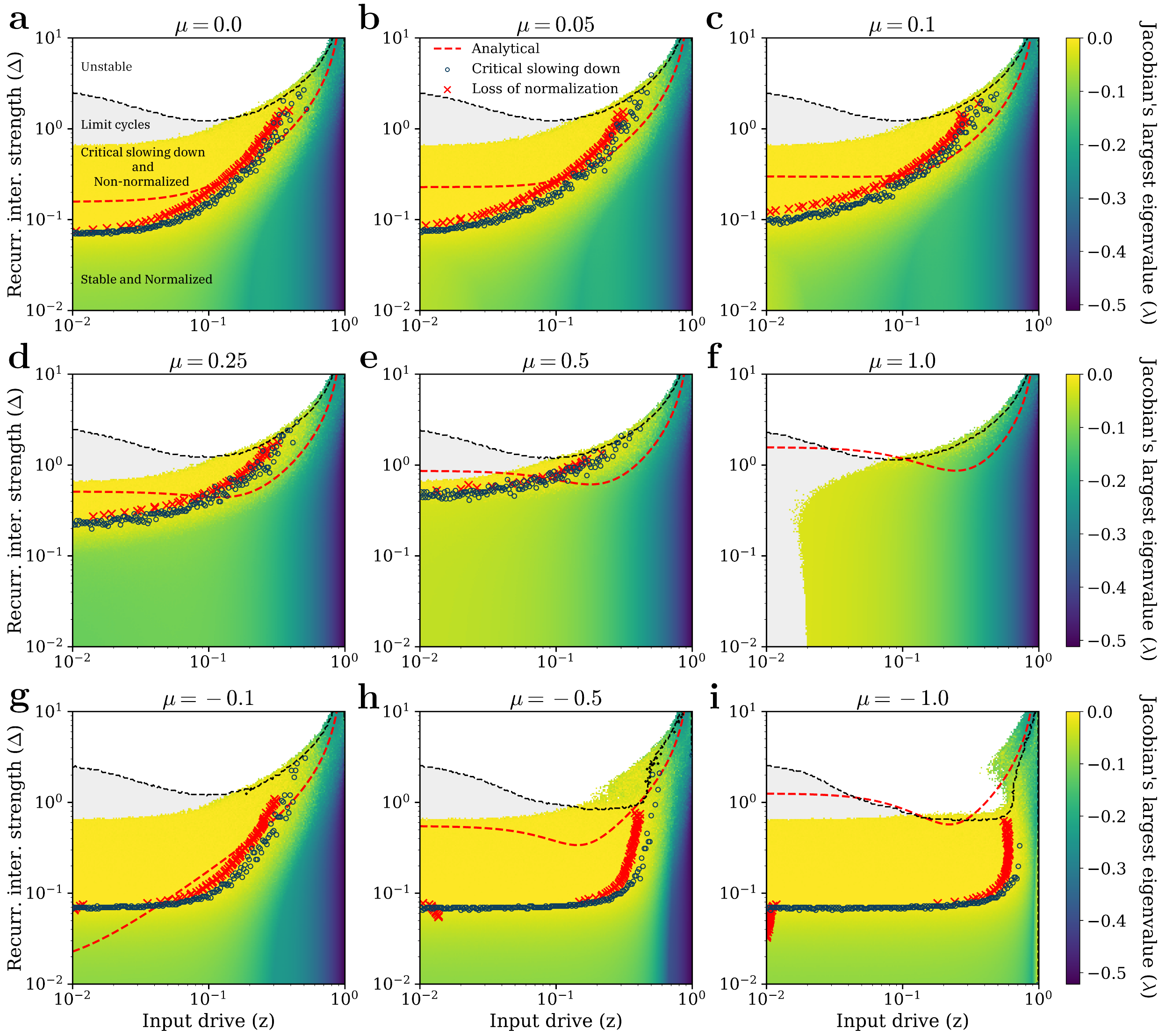

FIG. S11. **Effect of E-I imbalanced recurrence on stability.** Phase diagrams showing the real part of the largest eigenvalue λ of the Jacobian at the fixed point in the (z, Δ) plane for varying levels of E-I imbalance, controlled by the mean μ of the recurrent weights K_{ij}. Each panel corresponds to a different value of μ (see discussion in the text): **a,** $\mu = 0.0$ (balanced, identical to Fig. 5); excess excitation: **b,** $\mu = 0.05$, **c,** $\mu = 0.1$, **d,** $\mu = 0.25$, **e,** $\mu = 0.5$, **f,** $\mu = 1.0$; excess inhibition: **g,** $\mu = -0.1$, **h,** $\mu = -0.5$, **i,** $\mu = -1.0$. Color represents the maximum λ across 100 trials (for $N = 100$, $\sigma = 0.1$, $\tau_y = \tau_a$, delocalized input $z_i = z/\sqrt{N}$). Blue open circles mark the numerically determined onset of critical slowing down, while red crosses indicate the numerically determined loss of normalization. The dashed red curves show the analytical prediction for loss of normalization. Dashed black curves delineate boundaries between the limit cycle (gray region) and the unstable (white region) regimes, where instability is defined as trajectories diverging in at least 50% of trials.

bioRxiv preprint doi: https://doi.org/10.1101/2025.05.16.654567; this version posted May 21, 2025. The copyright holder for this preprint (which was not certified by peer review) is the author/funder, who has granted bioRxiv a license to display the preprint in perpetuity. It is made available under aCC-BY 4.0 International license.

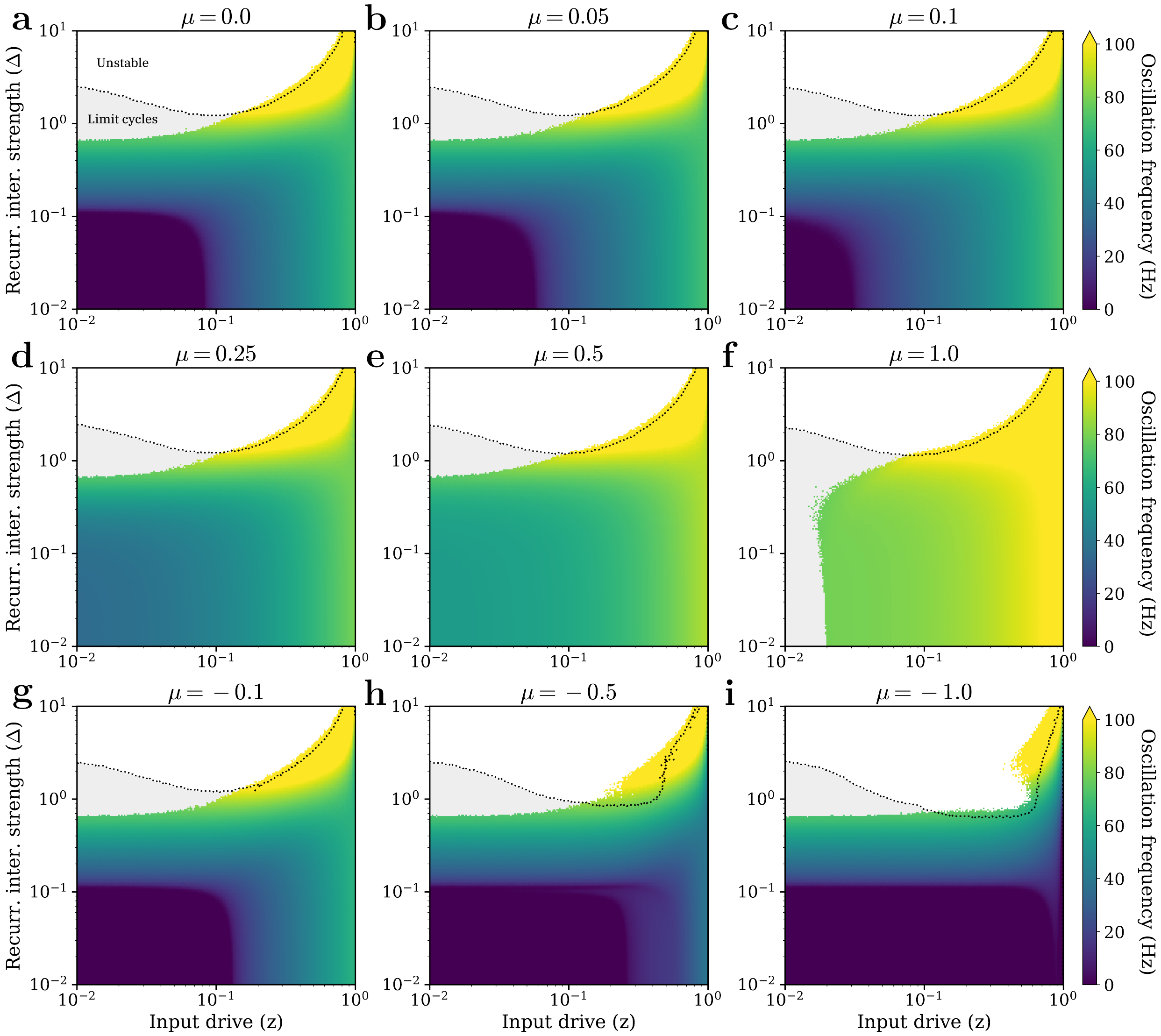

FIG. S12. **Effect of E-I imbalanced recurrence on oscillation frequency.** Phase diagrams depicting the average oscillation frequency (calculated as mean of $\mathrm{Im}(\lambda_{\mathbf{J}})/(2\pi)$ across trials) as a function of input drive z and recurrent interaction strength Δ for varying levels of E-I imbalance μ. Parameters are $N = 100$, $\sigma = 0.1$, $\tau_y = \tau_a = 2$ msec, delocalized input $z_i = z/\sqrt{N}$. Panels correspond to: **a,** $\mu = 0.0$; excess excitation: **b,** $\mu = 0.05$, **c,** $\mu = 0.1$, **d,** $\mu = 0.25$, **e,** $\mu = 0.5$, **f,** $\mu = 1.0$; excess inhibition: **g,** $\mu = -0.1$, **h,** $\mu = -0.5$, **i,** $\mu = -1.0$. Dotted black curves delineate boundaries between the limit cycle (gray region) and the unstable (white region) regimes, where instability is defined as trajectories diverging in at least 50% of trials. Overall, increasing excitation ($\mu > 0$) generally promotes higher oscillation frequencies, especially at small input drives. While increasing inhibition ($\mu < 0$) promotes lower oscillation frequencies, especially at large input drives.

bioRxiv preprint doi: https://doi.org/10.1101/2025.05.16.654567; this version posted May 21, 2025. The copyright holder for this preprint (which was not certified by peer review) is the author/funder, who has granted bioRxiv a license to display the preprint in perpetuity. It is made available under aCC-BY 4.0 International license.

G. Effect of critical slowing down on neural variability

A key marker of critical slowing down is a drastic increase in trial-to-trial neural variability and noise correlations between neurons. To illustrate why this occurs, we consider the ORGaNICs model with additive Gaussian white noise in the dynamical system:

$$\begin{cases} \tau_y \dot{y}_i = -y_i + z_i + (1 - a^+) \sum_{j=1}^{N} W_{ij} y_j + \sigma \eta_i(t) \\ \tau_a \dot{a} = -a + \sigma_{ss}^2 + \left(\sum_{i=1}^{N} y_i^+ \right) a + \sigma \eta_a(t) \ , \end{cases} \tag{40}$$

where $\eta_i(t)$ and $\eta_a(t)$ represent uncorrelated Gaussian white noise processes with zero mean and unit variance (i.e., $\mathbb{E}[\eta_k(t)\eta_l(s)] = \delta_{kl}\delta(t-s)$), and σ here denotes the strength of this noise. Note that σ_{ss}^2 is used for the semisaturation constant to avoid confusion with the noise strength σ. This introduction of stochasticity is distinct from the randomness in the recurrent matrix W considered in the main body of the manuscript.

Assuming the dynamical system operates in the vicinity of the stable fixed point (found in both stable and critically slowed-down regimes) and that the noise strength σ is sufficiently small, we can linearize the system around the fixed point. Let $\mathbf{x}$ be the vector of deviations from the fixed point. The linearized system is:

$$\dot{\mathbf{x}}(t) = \mathbf{J}\mathbf{x}(t) + \sigma \boldsymbol{\eta}(t) \ , \tag{41}$$

where $\mathbf{J}$ is the Jacobian matrix evaluated at the fixed point, and $\boldsymbol{\eta}(t)$ is the vector of white noise processes. The steady-state covariance matrix $\mathbf{P} = \mathbb{E}[\mathbf{x}\mathbf{x}^\top]$ (whose diagonal entries capture trial-to-trial variability and off-diagonal entries capture noise correlations) is given by the solution to the continuous-time Lyapunov equation:

$$\mathbf{J}\mathbf{P} + \mathbf{P}\mathbf{J}^\top + \sigma^2 \mathbf{I} = \mathbf{0} \ , \tag{42}$$

where $\mathbf{I}$ is the identity matrix, and $\sigma^2\mathbf{I}$ is the covariance matrix of the noise term $\sigma\boldsymbol{\eta}(t)$.

Assuming that $\mathbf{J}$ is diagonalizable, we can write its eigendecomposition as $\mathbf{J} = \mathbf{V}\boldsymbol{\Lambda}\mathbf{V}^{-1}$, where $\boldsymbol{\Lambda}$ is a diagonal matrix whose entries λ_i are the eigenvalues of $\mathbf{J}$, and $\mathbf{V}$ is the matrix whose columns are the corresponding eigenvectors. We can transform the coordinates to the eigenbasis of $\mathbf{J}$ by defining $\mathbf{y} = \mathbf{V}^{-1}\mathbf{x}$. The covariance matrix of $\mathbf{y}$ is $\mathbf{M} = \mathbb{E}[\mathbf{y}\mathbf{y}^\top] = \mathbf{V}^{-1}\mathbf{P}(\mathbf{V}^{-1})^\top = \mathbf{V}^{-1}\mathbf{P}\mathbf{V}^{-\top}$. Left-multiplying Eq. (42) by $\mathbf{V}^{-1}$ and right-multiplying by $\mathbf{V}^{-\top}$, we obtain:

$$\mathbf{V}^{-1}\mathbf{J}\mathbf{V}(\underbrace{\mathbf{V}^{-1}\mathbf{P}\mathbf{V}^{-\top}}_{\mathbf{M}}) + (\underbrace{\mathbf{V}^{-1}\mathbf{P}\mathbf{V}^{-\top}}_{\mathbf{M}})\mathbf{V}^\top\mathbf{J}^\top\mathbf{V}^{-\top} + \sigma^2\mathbf{V}^{-1}\mathbf{V}^{-\top} = \mathbf{0} \tag{43}$$

bioRxiv preprint doi: https://doi.org/10.1101/2025.05.16.654567; this version posted May 21, 2025. The copyright holder for this preprint (which was not certified by peer review) is the author/funder, who has granted bioRxiv a license to display the preprint in perpetuity. It is made available under aCC-BY 4.0 International license.

Using $\mathbf{V}^{-1}\mathbf{J}\mathbf{V} = \mathbf{V}^{\top}\mathbf{J}^{\top}\mathbf{V}^{-\top} = \mathbf{\Lambda}$ and defining $\mathbf{B} = \mathbf{V}^{-1}\mathbf{V}^{-\top}$, Eq. (43) becomes:

$$\mathbf{\Lambda}\mathbf{M} + \mathbf{M}\mathbf{\Lambda} + \sigma^2\mathbf{B} = \mathbf{0} \ . \tag{44}$$

This is the Lyapunov equation for the transformed coordinates $\mathbf{y}$. Since $\mathbf{\Lambda}$ is diagonal, we can solve for the entries of $\mathbf{M}$ element-wise:

$$M_{ij}(\lambda_i + \lambda_j) + \sigma^2 B_{ij} = 0 \implies M_{ij} = -\frac{\sigma^2 B_{ij}}{\lambda_i + \lambda_j} \ . \tag{45}$$

Therefore, if the real part of an eigenvalue, say $\mathrm{Re}(\lambda_k)$, approaches zero (which characterizes critical slowing down), the denominator $2\mathrm{Re}(\lambda_k)$ for the diagonal term M_{kk} becomes very small. Assuming B_{kk} (which depends on the eigenvectors) is non-zero, M_{kk} will become very large:

$$M_{kk} = -\frac{\sigma^2 B_{kk}}{2\lambda_k} \ . \tag{46}$$

As $\mathrm{Re}(\lambda_k) \to 0$, the magnitude of M_{kk} tends to infinity. Since the original covariance matrix entries P_{mn} are linear combinations of M_{ij} (as $\mathbf{P} = \mathbf{V}\mathbf{M}\mathbf{V}^{\top}$), a large M_{kk} will typically lead to large entries P_{mn}. This implies increased trial-to-trial variability (large diagonal elements of $\mathbf{P}$) and large noise correlations (large off-diagonal elements of $\mathbf{P}$) when the system is in the critically slowed-down regime compared to the stable and normalized regime.
